# Blood-based molecular and cellular biomarkers of early response to neoadjuvant PD-1 blockade in patients with non-small cell lung cancer

**DOI:** 10.1186/s12935-024-03412-3

**Published:** 2024-06-29

**Authors:** Xi Zhang, Rui Chen, Zirong Huo, Wenqing Li, Mengju Jiang, Guodong Su, Yuru Liu, Yu Cai, Wuhao Huang, Yuyan Xiong, Shengguang Wang

**Affiliations:** 1 School of Life Science, Northwest University, Xi’an, Shaanxi 710069 China; 2 Key Laboratory of Resource Biology and Biotechnology in Western China, Ministry of Education, School of Medicine, Northwest University, 710069 Shaanxi Xi’an, China; 3 Department of Lung Cancer, Key Laboratory of Cancer Prevention and Therapy, Tianjin Medical University Cancer Institute and Hospital, National Clinical Research Center for Cancer, Tianjin’s Clinical Research Center for Cancer, Tianjin Lung Cancer Center, Tianjin, 300060 China

**Keywords:** Anti-PD-1 blockade, Predictive biomarker, Early therapy response, Germline mutations, Immune cell subsets, Non-small cell lung cancer

## Abstract

**Background:**

Despite the improved survival observed in PD-1/PD-L1 blockade therapy, a substantial proportion of cancer patients, including those with non-small cell lung cancer (NSCLC), still lack a response.

**Methods:**

Transcriptomic profiling was conducted on a discovery cohort comprising 100 whole blood samples, as collected multiple times from 48 healthy controls (including 43 published data) and 31 NSCLC patients that under treatment with a combination of anti-PD-1 Tislelizumab and chemotherapy. Differentially expressed genes (DEGs), simulated immune cell subsets, and germline DNA mutational markers were identified from patients achieved a pathological complete response during the early treatment cycles. The predictive values of mutational markers were further validated in an independent immunotherapy cohort of 1661 subjects, and then confirmed in genetically matched lung cancer cell lines by a co-culturing model.

**Results:**

The gene expression of hundreds of DEGs (FDR *p* < 0.05, fold change < -2 or > 2) distinguished responders from healthy controls, indicating the potential to stratify patients utilizing early on-treatment features from blood. PD-1-mediated cell abundance changes in memory CD4 + and regulatory T cell subset were more significant or exclusively observed in responders. A panel of top-ranked genetic alterations showed significant associations with improved survival (*p* < 0.05) and heightened responsiveness to anti-PD-1 treatment in patient cohort and co-cultured cell lines.

**Conclusion:**

This study discovered and validated peripheral blood-based biomarkers with evident predictive efficacy for early therapy response and patient stratification before treatment for neoadjuvant PD-1 blockade in NSCLC patients.

**Supplementary Information:**

The online version contains supplementary material available at 10.1186/s12935-024-03412-3.

## Introduction

In the past decade, given the significant benefits achieved by immune checkpoint inhibitors (ICIs) in cancer, immunotherapy has emerged as a “common denominator” [1]. It has been demonstrated that combining anti-programmed death-1 (anti-PD-1) agents with chemotherapy can restore anti-tumor activities in multiple immune cell subsets, leading to increased overall survival [2]. Despite these impressive successes, the clinical benefit of this treatment remains limited to a small subset of patients [3]. Advanced NSCLC has been one of the first pioneers in becoming a common therapeutic focus for therapies targeting programmed death-1 (PD-1) or its ligand programmed death-ligand 1 (PD-L1) [4, 5]. The combination of anti-PD-1 therapy with chemotherapy has shown more encouraging results in the upfront treatment of NSCLC [6], although the overall response rate remains low. Taking the anti-PD-1 antibody Pembrolizumab as an example, the objective response rate for the unselected NSCLC population was only 19%, and the median overall survival was 12 months [7].

Numerous clinical studies have suggested that the detection of PD-L1 expression or tumor mutation burden (TMB) should serve as a companion diagnostic (CDx) assay for individuals newly diagnosed with NSCLC. [8–10]. Indeed, a few drug-specific companion diagnostic (CDx) tests have been approved to guide individualized anti-PD-1 treatment strategies for NSCLC patients [11, 12]. A recent guideline was just published by The American Society of Clinical Oncology (ASCO) that recommends patients across many cancer types should take germline genetic test [13]. Various molecular or cellular biomarkers with predictive efficacy for immune checkpoint inhibitors (ICIs) response have been suggested, encompassing gene expression biomarkers [14], tumor-infiltrating CD8-T cells [15], local or peripheral immune cell clusters [16, 17] and mutational DNA markers [18]. Pioneering studies in recent times put forth this hypothesis that the response of modern combination therapy is likely modulated by an intricate tumor ecosystem comprising diverse biological parameters which should be integrated in the development of predictive models for therapy response [19, 20].

In this study, a comprehensive analysis workflow was formulated to identify gene expression change, immune cell subset and germline mutation biomarkers that can predicting response of synergistic effect of immunotherapy and chemotherapy through transcriptomic analysis of a discovery cohort, followed by validation in a larger and independent cohort. By utilizing widely acknowledged computational tools and in vitro cell culture models, these markers also underwent extensive validations by published datasets, clinical evidences and genetically matched lung cancer cell lines. This collective approach enabled the identification of novel blood-derived biomarkers with the potential to guide combined therapy for NSCLC patients.

## Results

### Responder DEGs represent potential biomarkers from pre-therapy blood samples

**Fig. 1:**
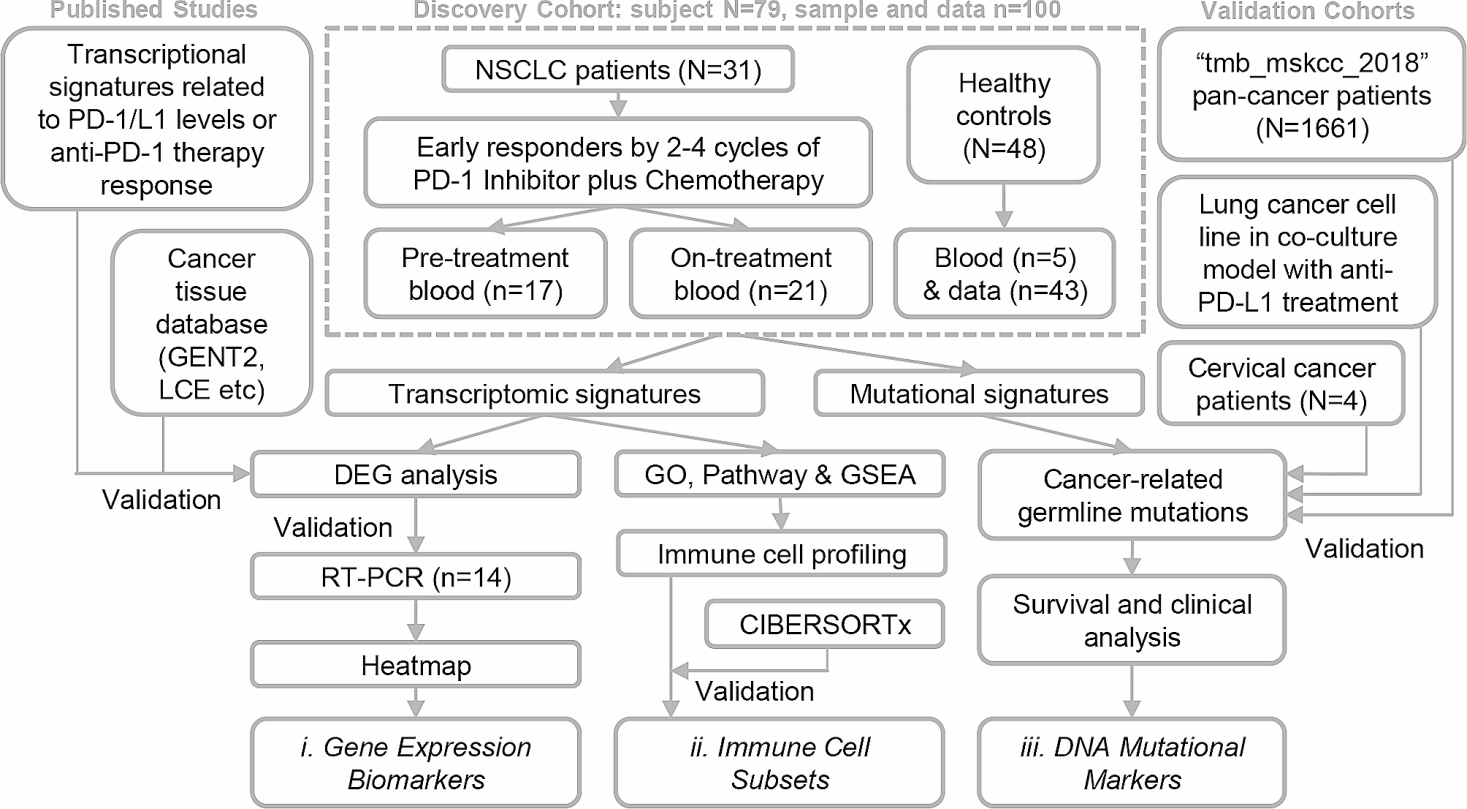
Workflow diagram of the study to identify blood-based signatures. The discovery cohort comprises a total of 100 blood samples collected from 79 subjects, including 43 subjects’ data obtained from publicly available database (GEO). NSCLC, non-small cell lung cancer; vs., versus; DEGs, differently expressed genes

**Fig. 2:**
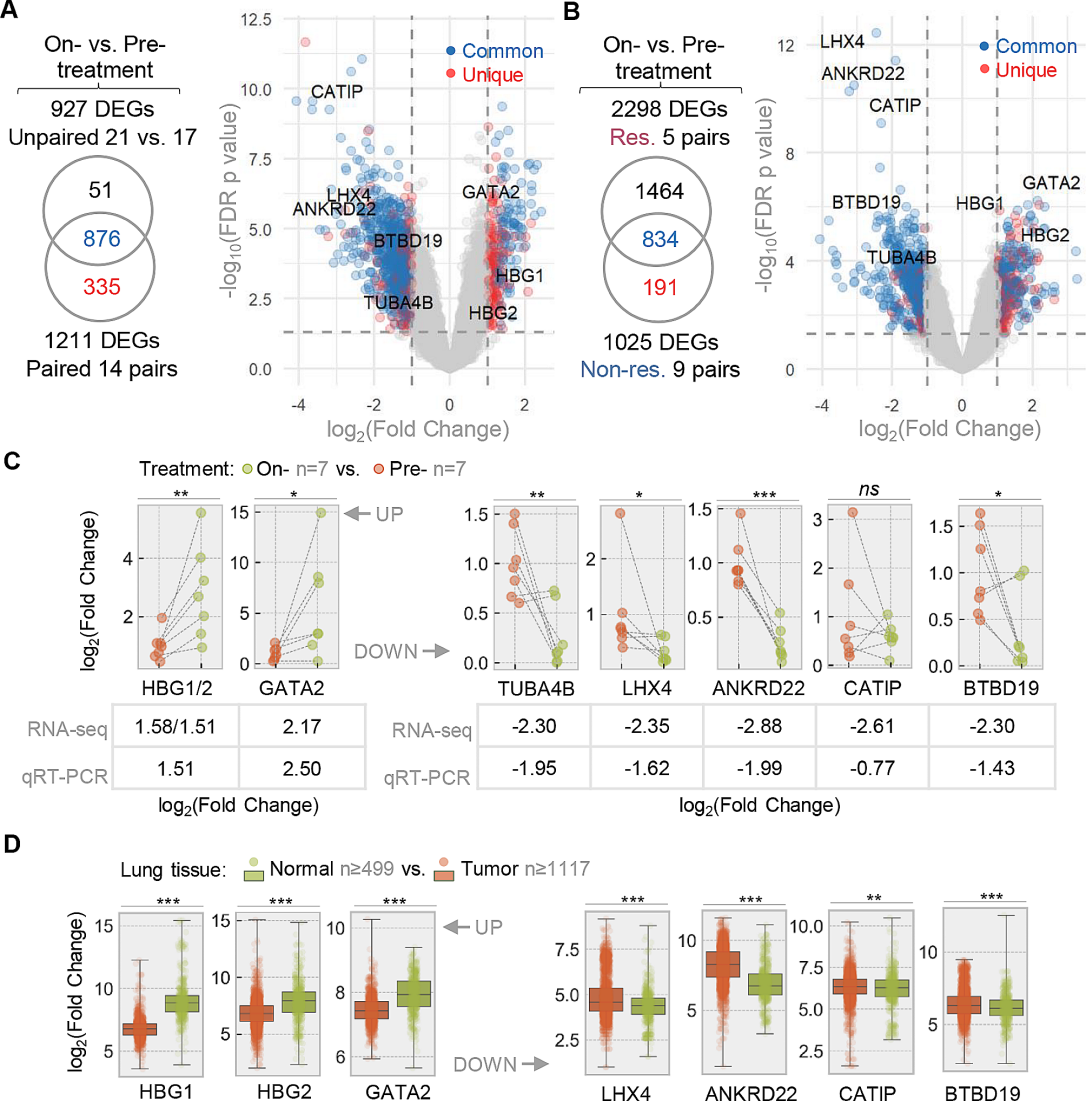
Identification and validation of transcriptomic signatures altered during neoadjuvant anti-PD-1 treatment. **A** & **B**) Venn diagrams and volcano plots of DEGs identified in overall comparisons **(A)** of on- versus pre-treatment blood samples and in individual comparisons between responder and non-responder subgroups **(B)**. Shared DEGs (Common) identified from unpaired (upper) and pairwise (lower) comparisons and DEGs only seen (Unique) in pairwise comparison are color-coded and plotted **(A)**. Common DEGs seen in responder (upper) and non-responder (lower) subgroups and Unique DEGs from non-responders are color-coded and plotted **(B)**. Expression changes of eight genes as annotated in volcano plots were confirmed by qRT-PCR. **C**) Relative mRNA expressions of 8 Common DEGs validated by qRT-PCR and compared to RNAseq results. The mean fold changes identified from both methods are provided after binary logarithmic conversion (Log_2_(mean fold change)). HBG1 and HGB2 were detected by same primer set. **D**) Relative mRNA expression changes in 8 Common DEGs in normal and lung cancer tissues (GENT2 database). vs., versus; DEGs, differently expressed genes; Res, responders; Non-res, non-responders; *, *p* < 0.05; **, *p* < 0.01; ***, *p* < 0.001

As described in Method (Fig. 1), a discovery cohort was recruited to collect blood samples from NSCLC patients what were under treatment of anti-PD-1 plus chemotherapy. We firstly identified 876 significant DEGs (FDR *p* < 0.05 and fold change > 2 or < -2) from unpaired and paired comparisons between on- vs. pre-treatment blood samples (Fig. 2A, Table S1, Fig. S1B). On top of the shared DEGs (*n* = 834), a larger number of DEGs were exclusively identified from responders (*n* = 1464, defined in Table S1) comparing to those only seen in non-responders (*n* = 191) (Fig. 2B, Figs. S1B & S1C). High or middle ranked DEGs were re-tested by Quantitative real-time PCR (qRT-PCR), showing consistent expression changes comparing to RNA sequencing (RNAseq) (Fig. 2C). In an independent tissue microarray database (GENT2), most of the representative genes (7/8) displayed consistent alteration (*p* < 0.01), comparing the differences from tumor to normal lung tissue versus the early on-treatment changes in blood samples (Fig. 2D). This result suggests an interesting agreement of between therapy-induced DEGs in blood and tumor-specific genes in tissue, which is further supported by results from another large lung cancer database (LCE) with meta-analysis across multiple independent cohorts (Fig. S2).

**Fig. 3:**
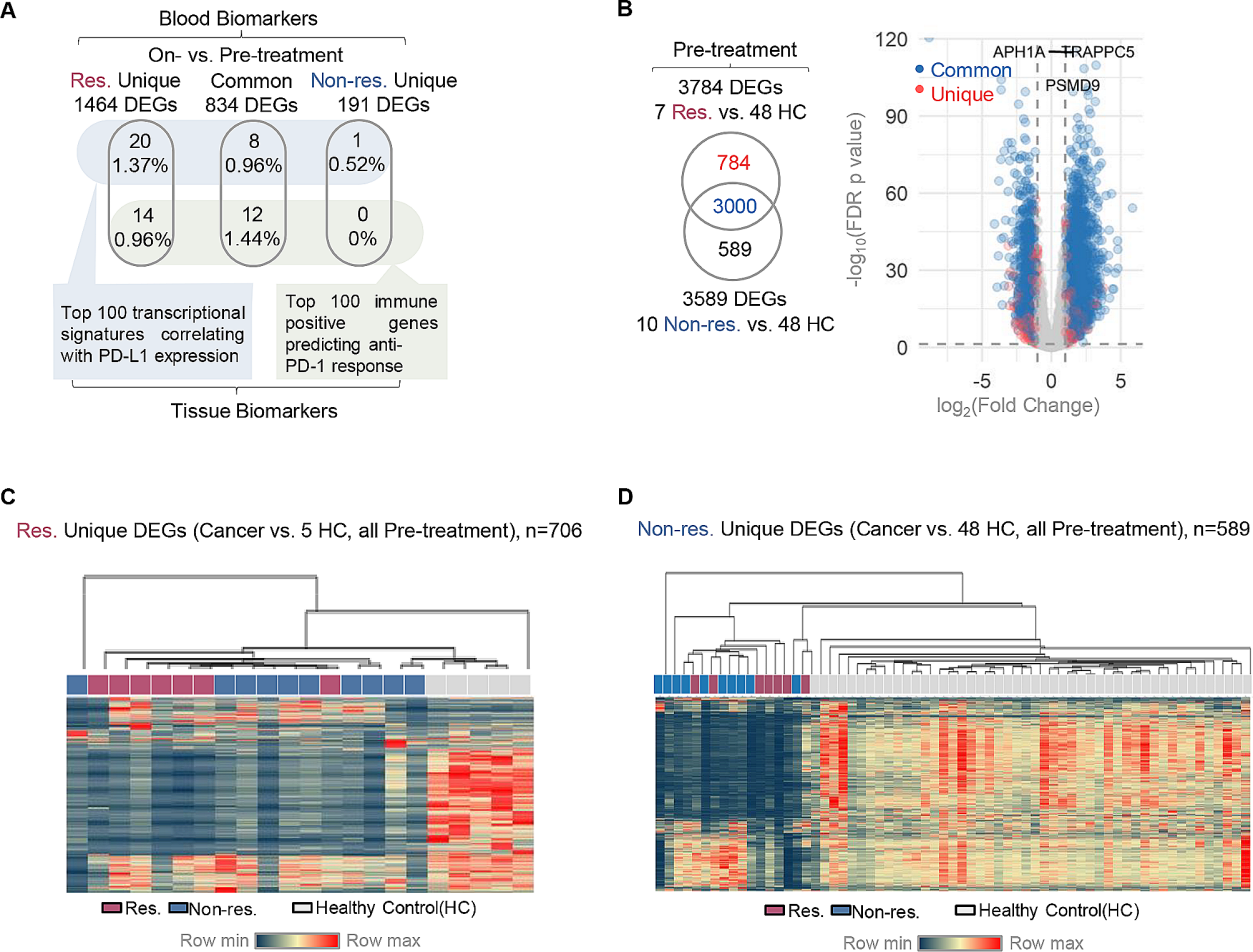
Characterization of pre-treatment blood biomarkers for patient stratification. **(A)** Venn diagrams showing the overlap between blood-derived DEG subgroups with published tissue-based molecular signatures, including the top-ranked cancer transcriptional signatures related with PD-L1 expression (blue box) and the gene expression signatures responding to anti-PD-1 therapy in melanoma (green box). **(B)** Venn diagrams and volcano plots of DEGs identified in comparing pre-treatment blood samples of responder and non-responder to healthy control (HC), respectively. Shared DEGs (Common) identified from both comparisons and DEGs only seen (Unique) in responders versus HCs are color-coded and plotted. **(C)** The hierarchical clustering of all pre-treatment samples according to the expression profiles of responder-specific Unique DEGs as identified comparing cancer versus healthy control (5 HC) samples. The heatmap visualized the relative expression level of each DEG. Sample status (healthy control, responder etc.) are color-coded and annotated. **(D)** The hierarchical clustering of all pre-treatment samples according to the expression profiles of non-responder-specific Unique DEGs as identified comparing cancer versus healthy control (48 HC) samples. The heatmap visualized the relative expression level of each DEG. Sample status (healthy control, responder etc.) are color-coded and annotated. vs., versus; DEGs, differently expressed genes; Res, responders; Non-res, non-responders; HC, healthy control

Next we sought to test our hypothesis that the responder-specific DEGs may serve as gene expression biomarkers to predict early therapy response. We started by investigating DEGs changed between on-and pre-treatment samples. Firstly our blood-derived DEGs were compared with two published tissue-derived transcriptional signatures that either correlates with PD-L1 expression [21] or responds to anti-PD-1 therapy in cancer [14]. It was observed the responder-specific DEGs (Unique DEGs) or the DEGs shared (Common DEGs) align better with the published signatures as compared to the non-responder-specific DEGs (Fig. 3A). However, the Unique DEGs among responders (Fig. S3A), non-responses and the Common DEGs (not shown) both prove ineffective in distinguishing between responder and non-responder samples, irrespective of their pre- or on-treatment conditions. Subsequently, we examined an additional set of DEGs that resulted from comparing patients’ pre-treatment samples with those of healthy individuals (healthy controls, HCs). A total of 784 and 589 significant Unique DEGs were generated in responders’ and non-responders’ blood, respectively (Fig. 3B & S3B, Table S1). It is noteworthy that the newly identified DEGs successfully distinguishes responders from non-responders, only using the pre-therapy blood samples (Fig. 3C and D). Correspondingly, we examined the DEGs that resulted from comparing patients’ on-treatment samples with those of healthy individuals (healthy controls, HCs). A total of 889 and 482 significant Unique DEGs were generated in on-treatment responders’ and non-responders’ blood, respectively (Figs. S3E & S3F, Table S1).

### Regulatory T cells form distinct cellular signatures in responders during the treatment

**Fig. 4:**
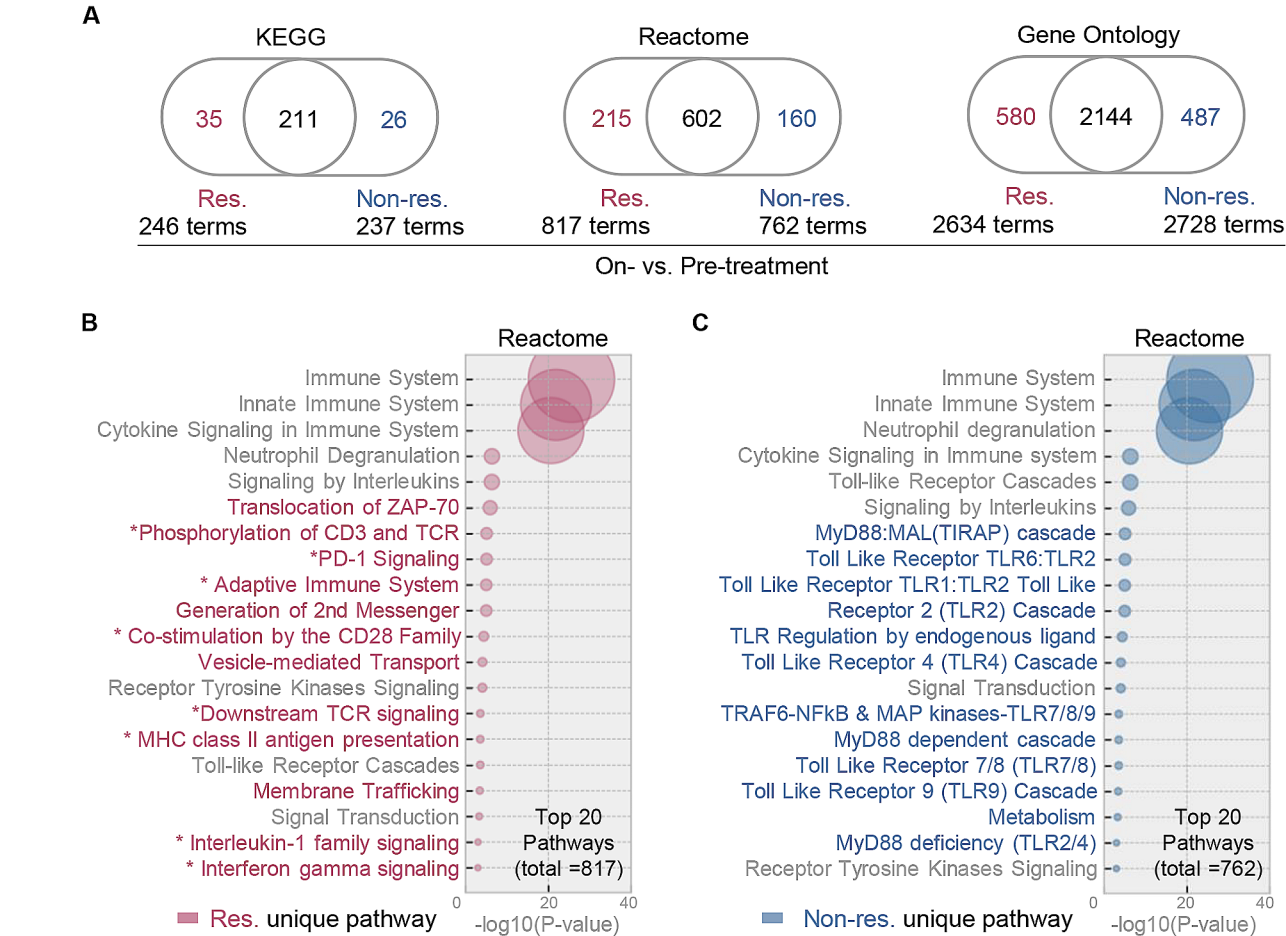
Characterization of pathways and GOs hat specifically regulated in responders. **A**) Venn diagram visualization of the significant KEGG pathways, Reactome pathways and gene ontology (GO) items regulated after treatment. The counts of terms regulated in responders, non-responders and both are provided respectively. **B** & **C**) Bubble plots of the top 20 pathways regulated in responders **(B)** and non-responders **(C)**. Bubble with bigger size stands for smaller *p* value and higher significance. The star denotes immune-related pathways. Names of unique terms are colored in red (responders), blue (non-responders) or grey (shared). Res, responders; Non-res, non-responders

**Fig. 5:**
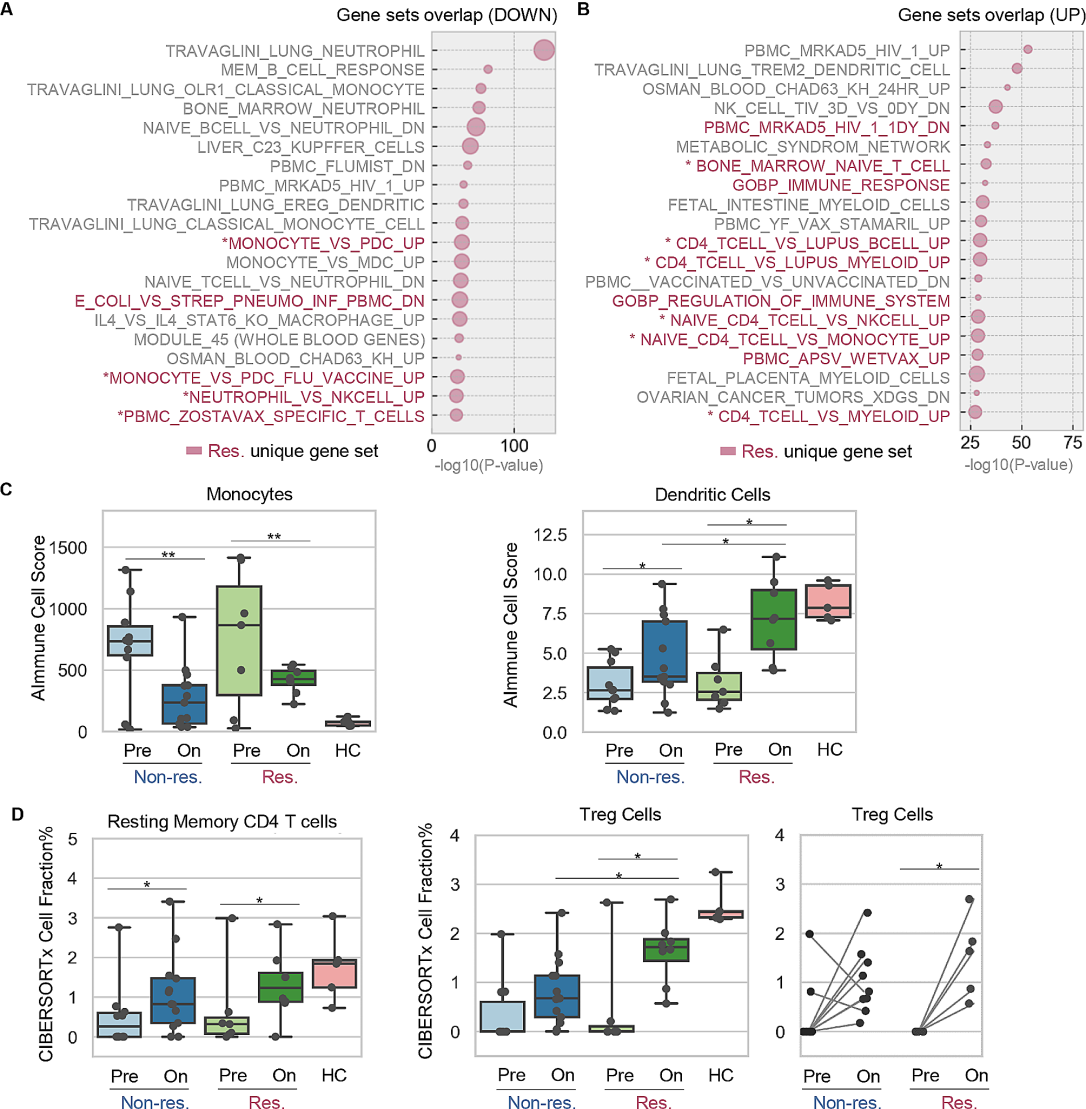
Identification of gene sets and immune cell subsets responding to the treatment. **A** & **B**) Bubble plots of the top 20 gene sets downregulated **(A)** and upregulated **(B)** in responders. Names of unique terms are colored in red (responders) while the shared terms are annotated in grey text. Bubble with bigger size stands for higher k/K value ratio and larger fraction of gene was matched with a certain reference gene set. The star denotes immune-related gene sets. **C** & **D**) Cell abundance scores as computed by AImmune **(C)** and CIBERSORTx **(D)** across responder and non-responder samples. Dot plots and box plots show individual values and average value of the scores. Connecting lines indicate the pairwise relationship between pre- and on-treatment samples. Res, responders; Non-res, non-responders; HC, healthy control; Pre, pre-treatment; On, on-treatment; *, *p* < 0.05; **, *p* < 0.01. All *p* values were calculated for pairwise comparisons

By employing the responder and non-responder unique DEGs identified above, a majority of pathway regulations were uncovered in all patients subsequent to administration of anti-PD-1 treatment (Fig. 4A, Figs. S4 & S5). On the contrary, crucial PD-1 signaling pathways were only significantly regulated in responders, including signaling of PD-1, CD3, TCR, CD28, IL-1 and IFN-γ (Fig. 4B and C). Consistent with this observation, gene set enrichment analysis (GSEA) suggested substantial alterations in immune cell subsets, including a decrease in monocytes and an increase in CD4 + T lymphocytes (Fig. 5A and B, Figs. S6 & S7). The immune cell abundance dynamics was further elucidated by *in silico* leukocyte deconvolution approach adopted from our previous work (AImmune) [22–24] and a classic machine learning tool CIBERSORTx [25]. These computing tools not only validated the changes in monocytes and resting memory CD4 + T cells but also unveiled an elevation in regulatory T cells, specifically observed in responders (Fig. 5C and D, Figs. S8 & S9).

### DNA mutations observed in responders are positively associated with enhanced survival

**Fig. 6:**
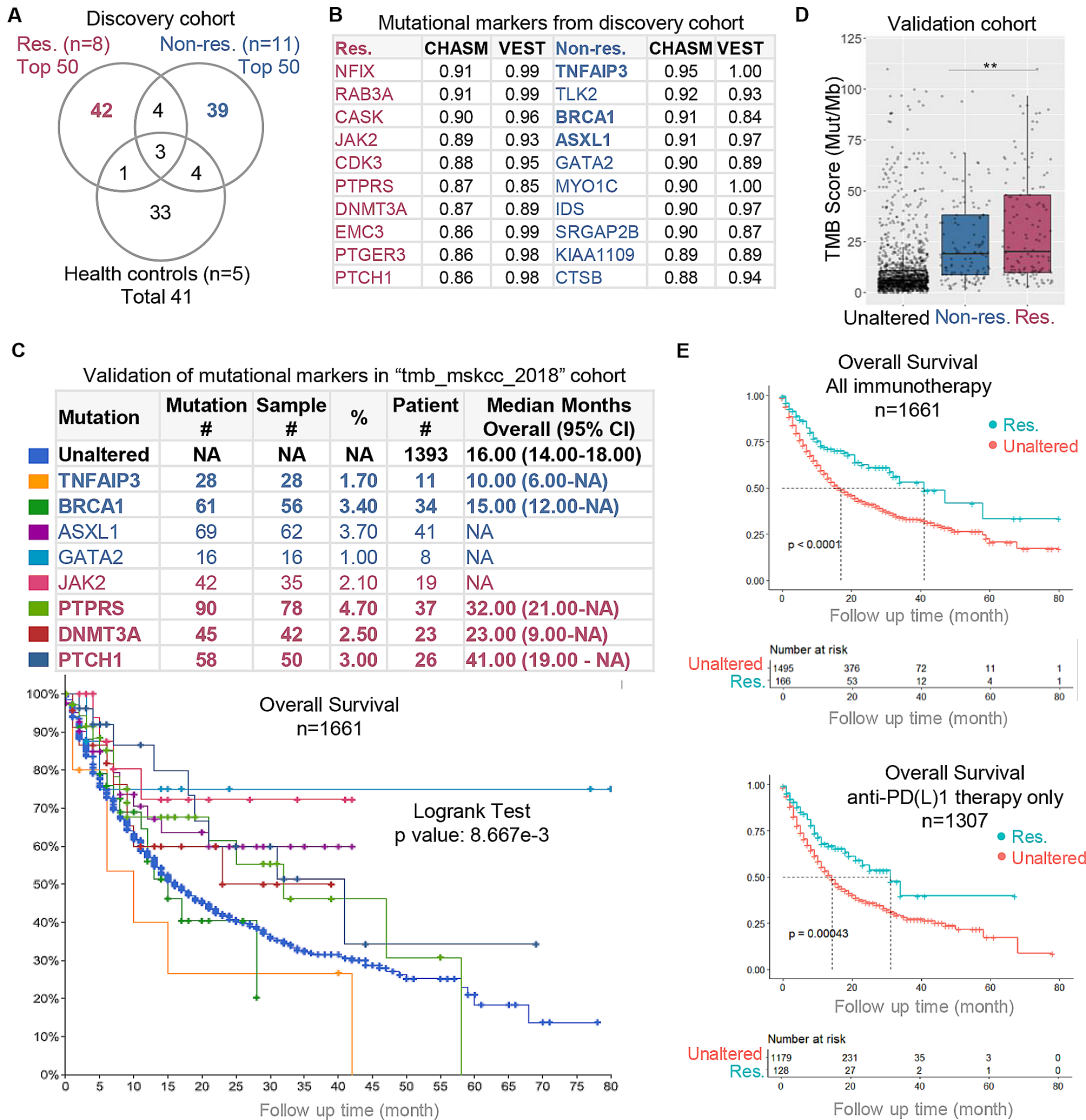
Identification and validation of DNA mutational markers in bloodstream. **(A)** Venn diagram showing cancer-related germline mutations as identified and ranked from discover cohort. **(B)** Top 10 mutated genes exclusively identified from responders and non-responders as ranked by CHASM and VEST scores. Mutations called in an independent cohort of with cervical cancer (Figure S11A) are indicated in bold. **(C)** Validation of the high-ranked mutations in an independent pan-cancer cohort (“tmb_mskcc_2018” cohort, *n* = 1661). Pan-cancer patients were stratified into 9 subgroups by the 8 mutational markers and their overall survivals were plotted by Kaplan-Meier curves. **(D)** Patients were stratified into 3 subgroups according to 2 mutational marker sets (4 for responders and 4 for non-responders) and their TMB scores were plotted. **(E)** Patients were stratified into 2 subgroups according to 1 mutational marker set (responder set). The Kaplan Meier overall survival curves of responder and unaltered subgroups as defined by mutational marker sets. Curves were generated for all immunotherapy patients (upper) and patients only received anti-PD(L)1 therapy (lower). *P* value was generated from by Log-Rank test which compares the survival distributions in individual groups as annotated. Res, responders; Non-res, non-responders; **, *p* < 0.01

The Cancer-Related Analysis of Variants Toolkit (CRAVAT) was employed in our discovery cohort to identify cancer-associated germline mutations that predict clinical benefit for anti-PD-1 treatment (Fig. S10). The top-ranked mutated genes (gene-level FDR *p* values < 0.05) were identified from responders, non-responders and healthy controls (Fig. 6A) and the top 10 genes from responders or non-responders are listed (Fig. 6B). The scores computed and ranked by CHASM (cancer driver classifier) and VEST (pathogenicity classifier) are all close to 1, suggesting confident classification of these germline variants as cancer-related mutations (Fig. 6B) [26, 27]. Three mutational markers (TNFAIP3, BRCA1, ASXL1) of non-responders were found to be shared with the DNA markers from patients with progressive disease in an independent cervical cancer cohort (GEO repository: GSE205247) (Fig. S11A). It is noteworthy that these three shared genes are ranked prominently as the top 1st, 3rd, and 4th mutations in the discovery cohort (bold in Fig. 6B).

In a considerably larger validation cohort (“tmb_mskcc_2018”) comprising 1661 pan-cancer patients [28], 8 out of the top 20 ranked gene mutations from the discovery cohort were detected in patients who all underwent PD-1 or cytotoxic T lymphocyte antigen 4 (CTLA-4) blockade treatment (Fig. 6C, upper). The Kaplan-Meier curves depicts a significantly difference of overall survival (*p* < 0.01) across 9 patient groups as defined by 8 mutational markers. Patients with no mutations show an overall median survival of 16 months, whereas those with non-responder mutations exhibit shorter median survivals (10–15 months), and patients with responder mutations demonstrate much longer median survivals (23–41 months), if available (Fig. 6C, lower). Subsequently, the patients were categorized into three subgroups using combined DNA mutations identified from responders (PTCH1, DNMT3A, PTPRS, JAK2) and non-responders (TNFAIP3, BRCA1, ASXL1, GATA2) as markers. TMB as the recognized favorable biomarker for clinical response to anti-PD(L)1 therapies, was observed to be highest in the responder subgroup (138 patients) and lowest in the unaltered patient subgroup (1307 patients) (Fig. 6D). As expected, the mean survival of the responder subgroup showed an extension compared to the unaltered patient subgroup seen in all patients (41 vs. 17 months) and only PD(L)1 blockade-treated patients (31 vs. 14 months) (Fig. 6E). The Cox regression results indicate hazard ratios of 0.559 (95% confidence interval: 0.425 to 0.735) for the responders in overall patient group and 0.579 (95% confidence interval: 0.425 to 0.789) in the PD(L)1 blockade only group. There was no significant difference observed when comparing the non-responder subgroup with the unaltered patient subgroup (Fig. S11).

### DNA mutational markers are validated in cancer cell lines co-cultured with immune cells

**Fig. 7:**
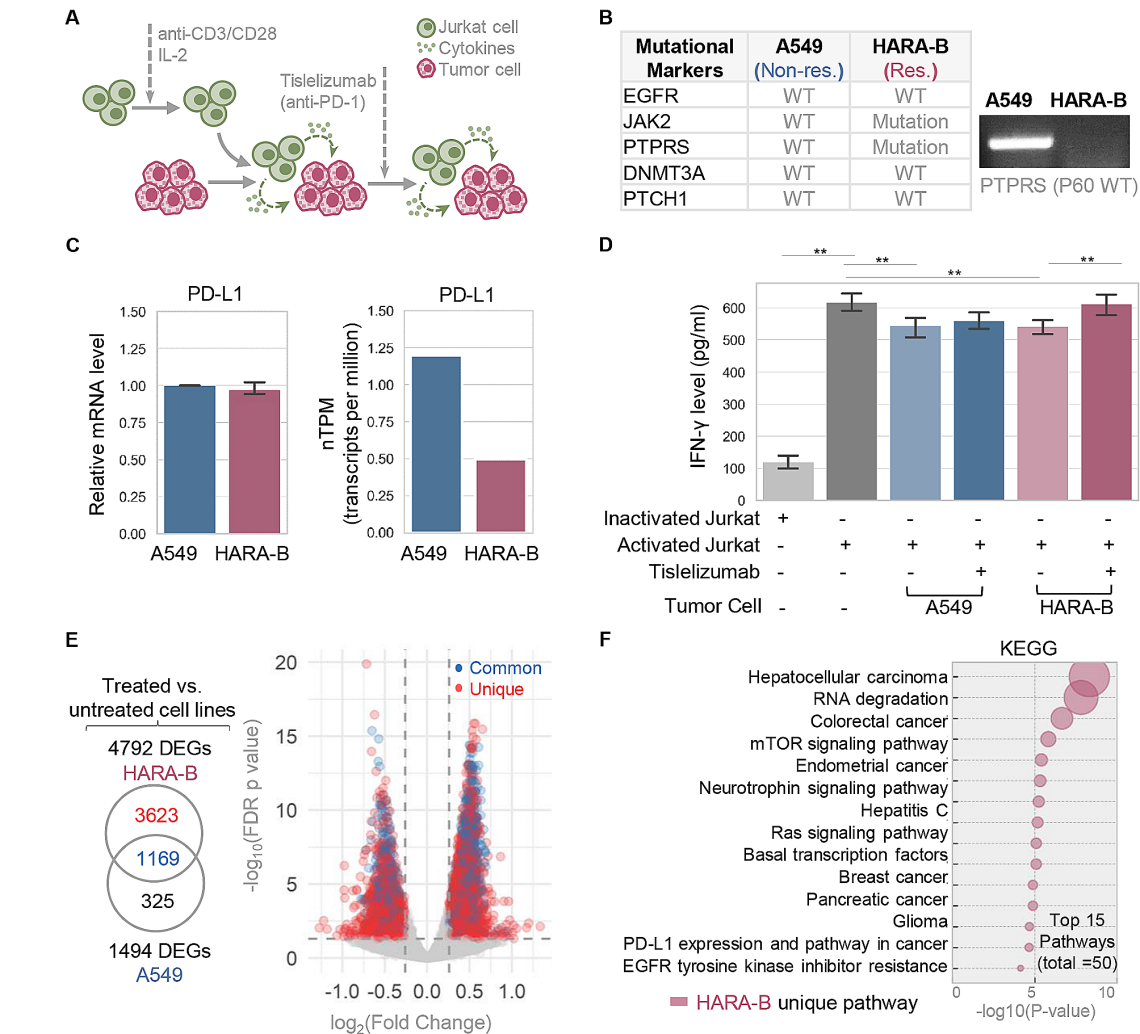
Evaluation of immunotherapy responsiveness of lung cancer cell lines carrying mutational markers. **(A)** Workflow diagram illustrating the co-culture model consisting of Jurkat cells and lung cancer tumor cells, which received anti-PD-1 treatment. **(B)** The genetic profiles of A549 and HARA-B cell line as obtained from the DepMap database (https://depmap.org/portal/) and confirmed by genotyping (PTPRS gene). **(C)** mRNA levels of PD-L1 in cell lines were assessed by qRT-PCR (left) and obtained from the Human Protein Atlas database (https://www.proteinatlas.org/) (right). **(D)** IFN-γ concentration in the supernatant of co-cultured cell lines was assessed by ELISA. **(E)** Venn diagrams and volcano plots of DEGs identified by comparing treated cells versus untreated cells. Shared DEGs (Common) identified from both cell lines and DEGs only seen (Unique) in HARA-B cell lines are color-coded and plotted. **(F)** Bubble plots of the top 15 downregulated KEGG pathways regulated in HARA-B cells. Bubble with bigger size stands for smaller *p* value and higher significance. DEGs, differently expressed genes; vs., versus. *, *p* < 0.05; **, *p* < 0.01. All *p* values were calculated for pairwise comparisons

The co-culture system involving activated immune cells and cancer cells is commonly utilized to acquire in-depth knowledge of immune-tumor interactions [29]. Here Jurkat CD4+-T-cell line was activated before co-culturing with lung cancer cell lines to assess their responsiveness to anti-PD-1 treatments (Fig. 7A). HARA-B and A549 cell lines, representing the molecular profiles of responder and non-responder respectively as identified earlier (Fig. 7B, left), were characterized before being studied pairwise in the co-culture model (Fig. 7B, right). HARA-B exhibited a similar (if not lower) PD‑L1 expression compared to A549 (Fig. 7C), aligning with their comparable immunosuppressive effects on IFN-γ production (Fig. 7D). The suppressed immune response was subsequently restored by the anti-PD-1 antibody Tislelizumab, as observed exclusively in HARA-B cells (responder) in contrast to the paired A549 cells (non-responder) (Fig. 7D). Indeed, transcriptomic proofing of treated versus untreated cell lines established that HARA-B cell but not A549 is highly sensitive to PD-1 inhibition. This is supported by the identification of a significantly larger number of DEGs in HARA-B (*n* = 3623) compared to A549 cells (Fig. 7E, Figs. S12A, Table S3). It is further proved by KEGG pathway analysis which revealed that cancer-driving signaling and PD-L1 signaling in HARA-B are prominently impaired, as marked by the down-regulated DEGs in the top-ranked pathways (Fig. 7F, Fig. S12B, Table S4). Based on these cell line data, it is likely that lung cancer cells carrying responder mutations would exhibit a more favorable response to treatment with PD-1 inhibitors.

## Discussion

The overarching hypothesis of this study is that blood-based signatures identified from early responders to tislelizumab plus chemotherapy would offer prognostic values. To clarify, three comparison strategies were employed to identify blood-derived biomarkers: (1) DEGs identified by comparing pre-treatment responders vs. pre-treatment non-responders were used to stratify responder patients before treatment (Fig. 3C and D); (2) DEGs identified by comparing changes from pre-treatment to on-treatment in responders vs. non-responders were utilized to characterize responsiveness-related pathways (Fig. 4B, pink) and immune cell subsets (Fig. 5), which are different from intervention-related pathways (Fig. 4B and C, gray) etc.; (3) DNA mutations identified in responders and non-responders (irrespective of pre-treatment or on-treatment status) indicate mutation markers for stratifying responder patients. Our findings offer new evidences suggesting gene expression signatures, peripheral immune cell clusters, and DNA mutational determinants that profiled through a blood draw may predict clinical efficacy either before the combined therapy or during the early treatment cycles.

The initiation of this study identified a set of Common DEGs that exhibited changes during early treatment. The significance of this findings lies in the fact that these DEGs were, on one hand, triggered by early therapy in blood samples, as confirmed by both RNAseq and qRT-PCR; on the other hand, their expression levels also changed consistently in tissue when comparing tumor to normal samples. One of the validated DEGs (HBG1) that was up-regulated in treated patients here, showed a continuous increase in advanced NSCLC patients during the 2nd to the 5th cycles of treatment [30]. Other validated DEG genes includes a hematopoietic transcription regulator (GATA2) [31], a metabolism mediator (ANKRD22) [32, 33], and a transcription factor involved in differentiation control (LHX4) [34]. This is consistent with the current understanding that immunological and metabolism mechanisms are enriched in patients received treatments [35]. The DEGs from early responders exhibited overlap with two sets of published signature genes identified from cancer tissue. One set is the top 100 (out of the total 1788) transcriptional correlates of PD-L1 expression [21] and the other set consists of 100 immune-positive genes utilized for predicting melanoma patient response [14]. In comparison to the first set of DEGs requiring blood samples from on-treatment patients, a second set of DEGs was identified from pre-therapy samples (responders vs. healthy controls) and exhibited promising biomarker features, offering better distinction of early responders from other subjects. Notably, the top-ranked genes in this list (Fig. 3B) are either previously reported as prognostic biomarkers (PSMD9 and APH1A) for other cancer indication (cervical cancer and HCC) [36, 37], or are known a vesicular trafficking modulator (TRAPPC4) that regulates the intracellular trafficking of PD-L1 and antitumor immunity [38]. Collectively, the predictive values of these newly identified markers are novel when obtained from the bloodstream of lung cancer patients during the early stages of treatment. Prior to this study, their significance had only been reported in other cancer indications or as therapeutic targets rather than as biomarkers.

PD-1 blockade therapy is known to crosstalk with T cell activation, differentiation, and other immune cell activities. There is a growing body of evidence confirming that the efficacy of chemotherapies also depends on activating antitumor immune responses. It is logical for us to observe PD-1 signaling and PD-1-regulated signaling cascades (such as CD3, TCR, CD28, IL-1 and IFN-γ) enriched only in responders. Our GSEA and immune cell abundance analysis provided additional insights into the orchestration of immune clusters, aligning with published findings. Firstly, it was expected that we found monocyte-to-DC differentiation to be significantly higher at the early stage of therapy for NSCLC patients, given this differentiation is known to attenuate CD8 + T cell response and predict clinical outcomes of patients with other cancers [39–41]. Secondly, our observation of enriched peripheral CD4 + T cell subset in responders is consistent with a recent study that characterized NSCLC patients who received anti-PD-1 therapy [42]. The uniqueness of our study lies in the approach, as RNAseq requires a minimal blood specimen compared to the flow cytometry as employed in by the existing study. At last, our method also revealed a significant elevation of regulatory T cell (Treg cell) in circulating blood of responders. This supports the prevailing understanding that PD-1 blockade facilitates the proliferation of highly suppressive PD-1 + Treg cells [43]. Given the complex and sometimes conflicting conclusions in this field, the prognostic value of peripheral immune cell subsets in anti-PD-1 therapy needs to be addressed through further fine-tuned studies.

Emerging studies as well as a recent ASCO guideline provide evidence-based recommendations that pathogenic germline variants can predict patient outcomes [13, 44, 45]. The present study has identified 4 cancer driver genes (PTCH1, DNMT3A, PTPRS, JAK2) that rank highest in responders and are linked to enhanced survival in the validation cohort, either as individual markers or as part of a grouped marker panel. Individual diver gene mutations were previously identified from tumor tissue demonstrated to promote (such as KRAS [46], TP53 [47], PTCH1 [48]) or weaken (such as JAK1/2 [49], EGFR [50], PTEN [51]) the response to immune checkpoint inhibitor therapy in cancer patients. The driver role of somatic mutation is consistent across cancer indications. An typical example is TNFAIP3 mutation indicates low responses to PD-1 inhibitor in NSCLC and cervical cancer patients as showed in the present study, which is also supported by a study on melanoma [52]. Another example is that the association of PTCH1 mutation with improved outcome of PD-1 blockade was seen in both colorectal cancer [48] and NSCLC (the present study). Improved responsiveness is also observed in human squamous cell lung carcinoma HARA-B, which harbors mutations in two marker genes, compared to the genetically matched control cell line A549. This occurs after coculturing with CD4 + Jurkat cell line and both undergoing treatment with PD-1 blockade. Patients with these mutational markers exhibit a higher tumor mutational burden (TMB), a well-established marker correlated with improved survival in NSCLC patients treated with PD-1 plus CTLA-4 blockade [53, 54]. While the prognostic values of these (or similar) genes with somatic mutations have been highlighted recently [48, 55–57], our study stands out as a unique research endeavor identifying germline mutations to be cancer-related and associated with increased susceptibility. Collectively, our results offer robust evidence affirming the predictive significance of these DNA mutational markers, even though they are detected in peripheral blood rather than tumor tissue.

While our study offers crucial insights into the biomarker features in bloodstream and the molecular mechanism of resistance in PD-1 blockade therapy plus chemotherapy, the discovery dataset is derived from a small cohort. Therefore, we aimed to validate our observations using large independent cohorts of pan-cancers that received similar treatments, as well as in genetically matched cell lines. It is important to note that the cell abundance analysis tool AImmune employed here is still in its early development stage in-house. Despite undergoing robust testing and validation in our published studies [22–24], the current version is limited to cover up to 10 major immune cell subsets from peripheral blood. Rare cell populations or the small-scale dynamics of immune cells might be neglected. For instance, changes in composition of PD-L1 + or CD14 macrophages and CD62L^low^ CD4 + T cells, as detected by flow cytometry, were emphasized in PD-1 blockade responders [58–60]. These cell subsets would need to be evaluated once a more finely tuned computational tool is available.

## Method

### Study cohorts and overall workflow

This study constructed a discovery cohort and collected a total of 100 whole blood samples (or data) from 5 healthy controls, in combination with another 43 published healthy blood samples (RNAseq data from Gene Expression Omnibus (GEO): GSE152641, GSE160351, GSE166253, GSE206263) [61–63] (Figs S3C, S3D), and 31 EGFR wild-type NSCLC patients visited Tianjin Cancer Hospital from 2020 to 2022 (Fig. 1; Table 1). All patients were ineligible for EGFR-targeted therapies (due to genotyping result) and thus underwent treatment by anti-PD-1 monoclonal antibody Tislelizumab [64, 65], in combination with standard chemotherapy. Early on-treatment clinical benefit was observed in 10 out of 31 patients, indicating a positive response. Among these responders, 7 had valid pre-treatment samples, while 8 had valid on-treatment samples. The majority of enrolled patients were male (87.1%), over 60 years old (64.5%), ever-smokers (80.6%), and diagnosed with lung squamous cell carcinoma (SqCC) (83.9%) (Table 1). The protocol was approved by the local Ethics Committee and the Institutional Review Board of Norwest University (approval number: 200,402,001) and all subjects provided written informed consent.

**Table 1:** General characteristics of NSCLC patients in this study (*N* = 31)

Characteristics	Count (percentage)
Gender	
Male	27 (87.1%)
Female	4 (12.9%)
Age	
≥ 60	20 (64.5%)
< 60	11 (35.5%)
Smoking history	
Yes	25 (80.6%)
No	6 (19.4%)
Histology	
Squamous (SqCC)	26 (83.9%)
Adenocarcinoma (ADC)	4 (12.9%)
Large cell carcinoma (LCA)	1 (3.2%)
Stage	
IA	10 (32.3%)
IB	4 (12.9%)
IIA	1 (3.2%)
IIB	5 (16.1%)
IIIA	5 (16.1%)
IIIB	3 (9.7%)
IVA	1 (3.2%)
Unable to stage	2 (6.5%)
Responder status	
Responder (pCR*)	12 (38.7%)
Non-responder (non-pCR)	17 (54.8%)
Not evaluated	2 (6.5%)

*pCR, pathologic complete response

Whole blood samples were collected twice: the first one up to one month before the initiation of therapy (pre-treatment), and the second one before completion of 2 to 4 cycles (on-treatment). Patients achieved pathological complete response (pCR) during this period were categorized as “responders” and those did not achieve pCR were labelled as “non-responders”. Specimens that failed during sampling process or did not meet RNAseq QC were excluded, resulting a total of 57 cancer and 5 healthy donor (healthy control) samples sequenced in the discovery cohort. The candidate molecular biomarkers were identified through RNAseq, validated by RT-PCR and then compared to known immunotherapy signatures from published clinical studies [14, 21] as well as lung cancer tissue datasets (GENT2: https://gent2.appex.kr; LCE: https://lce.biohpc.swmed.edu) (Fig. S1A). Pathways and cellular biomarkers were investigated via KOBAS-i portal (http://kobas.cbi.pku.edu.cn/) [66] and computational tools (AImmune; CIBERSORTx: https://cibersortx.stanford.edu). Cancer-associated germline mutations were called by CRAVAT (https://www.cravat.us/CRAVAT).

The findings of mutational markers were compared with an independent study (GEO: GSE205247) and then rigorously assessed in an independent validation cohort (tmb_mskcc_2018 from cBioPortal) [28, 67] (Fig. S1A). Finally, we tested the responsiveness of paired lung cancer cell lines that harboring mutational markers or wildtype genotypes after co-cultured with T lymphocyte cell line and treated by anti-PD-1 antibody.

### RNAseq, and qRT-PCR

PBMCs of 5 healthy control donors or cultured cell lines in 2 or 3 replicates were isolated from whole blood or cell pellets were collected at the desired end point of co-culture models. One PBMC samples was collected and sequenced for each patient at single or multiple timepoints (pre- or/and on-treatment). The total RNA was isolated using Trizol (Invitrogen, Carlsbad, CA, USA) and the purity and concentration were verified using a NanoDrop ND-1000 instrument (ThermoFisher Scientific, Waltham, MA, USA). The integrity of the RNA was assessed by a 2100 Bioanalyzer gel image analysis system (Agilent, Santa Clara, CA, USA) before transcriptomic analysis (RNAseq) and (or) Quantitative real-time PCR (qRT-PCR).

Qualified RNA samples were then enriched and synthesized into two strand cDNA for library preparation. RNAseq libraries were constructed using the TruSeq RNA Sample Prep Kit (Illumina, San Diego, CA, USA). The libraries from qualified RNA samples were sequenced in the 150 nt paired-end mode on an Illumina NovaSeq 6000 platform at Novogene Bioinformatic Technology (Tianjin, China). After quality filtering (FastQC, quality value > 5), over 30 billion clean reads were obtained in each library and then used for down-stream analysis. PCR primers were designed for selected genes as obtained by differential gene analysis (Table S2). Real-time quantitative PCR was performed in real-time PCR systems (Bio-Rad, Hercules, CA, USA). The relative expression levels were calculated by the 2^−ΔΔCt^ method. Two or three replicates were measured for each sample.

### Cell culture and co-culture model

Lung cancer cell lines (A549 and HARA-B) and Jurkat T (Jurkat) cell lines were purchased from Procell Life Science & Technology (Wuhan, Hubei, China). A549 cells were cultured in Dulbecco’s modified Eagle’s medium (DMEM, Procell, Wuhan, Hubei, China). HARA-B and Jurkat cells were cultured in RPMI-1640 (Procell, Wuhan, Hubei, China). In both media, 10% fetal bovine serum (FBS, Bioind, Israel) and 1% penicillin/streptomycin (Procell, Wuhan, Hubei, China) were added. The above cells were grown in a humidified 5% CO_2_ incubator at 37 °C.

The workflow of co-culture model is illustrated in Fig. 7A. Briefly, A549 and HARA-B cells were seeded onto 6-well plate at 1 × 10^5^ per ml and cultured in their respective required media (as described above). The Jurkat cells were pre-activated with 3 µg ml^− 1^ anti-CD3 (BioGems, Westlake Village, CA, USA), 2 µg ml^− 1^ soluble anti-CD28 (BioGems, Westlake Village, CA, USA) and IL-2 (25 ng ml^− 1^, Peprotech, Cranbury, NJ, USA) for 48 h. Briefly, we first diluted anti-CD3 in PBS and incubated overnight at 4 °C. After discarding the PBS, use fresh 1640 medium (including anti-CD28 and IL-2) to culture Jurkat cells in a 37 °C incubator for 48 h. Jurkat was then mixed with A549 and HARA-B cells at a density of 1 × 10^6^ per ml (the ratio of Jurkat cells to tumor cells is 10:1) and maintained in fresh RPMI-1640 medium containing 10% FBS, and all cells were treated with or without 50 µg ml^− 1^ anti-PD-1 antibody Tislelizumab (MCE, Monmouth Junction, NJ, USA) for 24 h.

### Enzyme-linked immunosorbent assay (ELISA) for detection of IFN-γ

Interferon-γ (IFN-γ) level in supernatants of the co-culture model was measured using Human IFN-γ Enzyme-linked immunosorbent assay kit (MLBIO, Shanghai, China) according to the manufacturer’s protocol. Optical density was measured at 450 nm, and the IFN-γ level was calculated from a standard curve prepared using the recombinant protein provided in the kit.

### Genotyping

DNA was extracted from lung cancer cells using Genomic DNA Extraction Kit (Tiangen, Beijing, China) followed by a standard PCR amplification with GoTaq Green master mix (Promega, Madison, WI, USA). Amplified DNA was separated and visualized by agarose gels (2%). The DNA bands were imaged using an automatic digital gel image analysis system (Tanon-1600, Shanghai, China).

### Computational analysis to quantify immune cell abundance

A novel *in silico* leukocyte deconvolution method, named AImmune, is a computational approach developed by integrating our established immune cell profiling [30] with published single cell RNAseq data obtained from NSCLC blood samples (1071 qualified cells from one patient, GSE127471) and healthy blood samples (8369 and 7687 cells from two donors, 10X Genomics). Briefly, with the additional marker genes included, more than 30 candidate marker genes for each cell subsets in peripheral immune cell subsets (CD4-T cells, CD8-T cells, B cells, Monocytes, DCs, NK cells and NKT cells) were selected based on their expression patterns across immune cell subsets [68]. The pairwise similarity statistic of all cell subsets was computed (data not shown) between all pairs of the candidate marker genes within the normalized RNAseq profiles (FPKM) from whole blood samples. Using the criteria (average Pearson correlation factor > 0.60, *p* < 0.01), 10–20 selected marker genes were identified as our final marker genes. The raw cell abundance score was calculated as the sum of the simple averages of the marker genes’ log2 expression, which allows comparison of cell composition across subject groups. This approach also tested a novel deconvolution model (unpublished) built by DNN (deep neural networks) algorithms and then trained by pseudo-bulk samples obtained by randomly subsampling of published single-cell RNA sequencing (scRNAseq) data [69]. Machine learning-based feature extraction (marker gene selection) was integrated for model optimization. Most of the computational analysis procedure was coded by common Python packages; scRNAseq data was processed by R package scanpy; machine learning model was developed and tested with Python library Tensorflow. All computational analysis were performed and visualized using R version 3.6.1 or Python version 3.7.9.

### Bioinformatics and variant calling

All raw RNAseq reads were filtered by R package trim_fastq to remove adapters, rRNA and low-quality reads. The QC criteria included: removing bases below Phred quality 20, containing over two “N”, or shorter than 75. The output reads were then indexed by aligner STAR and mapped to reference genome by BAM. Normalized read counts were generated and compared between groups to generate DEGs using R package DESeq2. Another R package countToFPKM was employed to produce FPKM for AImmune analysis. Genes in PBMC samples that displayed at least two-fold difference in gene expression between comparison groups (fold change > 2 or < -2, FDR *p* < 0.05) were considered significant differentially expressed genes (DEGs) and carried forward in the analysis. DEGs in lung cancer cell lines were identified by lower threshold (fold change > 1.2 or < -1.2, FDR *p* < 0.05) to maximin DEG count as illustrated in a volcano plot. Hierarchical clustering was performed to show the gene expression patterns and similarities among samples. Pathway and gene ontology (GO) enrichment analysis was carried out via an integrated platform KOBAS 3.0 [35]. GSEA analysis was carried out by searching the established MSigDB gene-set collections (C7). CIBERSORTx analysis was performed following the instruction from the portal (https://cibersortx.stanford.edu). Differences of mRNA levels and cell abundance scores were evaluated using independent *t*-tests or paired *t*-tests if pairwise samples were given.

Variant calling was performed using the HaplotypeCaller that plug-in in the Genome Analysis Toolkit v4.0 (GATK). First, RNA reads were aligned to the reference genome using the STAR aligner, then the MarkDuplicates was used to clean up data. The gatk BaseRecalibrator and gatk ApplyBQSR were used to adjust the mass fraction of original bases, detect the system errors in mass fraction, and reduce the false positives. We used only variants marked with PASS in the VCF file and filtered the variant calls with the VariantFiltration tool. The Cancer-Related Analysis of Variants Toolkit (CRAVAT) is a well-recognized informatics toolkit used in this study for variant calling from VCF files [70, 71]. This tool covers multi-level mutational analysis functions including mutation mapping and quality control, impact prediction and extensive annotation. Two Random Forest filters were employed in CRAVAT for predicting mutation impact, namely Cancer-Specific High-Throughput Annotation of Somatic Mutations (CHASM) and Variant Effect Scoring Tool (VEST). CHASM is a classifier that classifies if a mutation is an oncogenic driver while VEST rates if a mutation is pathogenic or benign.

A *p*-value of < 0.05, < 0.01, and < 0.001 was considered statistically significant, annotated by *, **, and ***, respectively. Kaplan-Meier analysis was utilized to estimate the survival curve of cancer patients and to calculate the incidence of each mutation subgroup over time. Additionally, a Cox regression model was conducted to quantitatively measure the hazard ratio of each subgroup. Bioinformatics and statistics analyses were performed and visualized using R version 3.6.1 or Python version 3.7.9.

### Electronic supplementary material

Below is the link to the electronic supplementary material.

Supplementary Material 1: figure S1 (A) Summary of validation cohorts, validation methods and additional datasets used in this study. (B) Venn diagrams and volcano plots of DEGs identified in overall comparisons (left) of on- versus pre-treatment blood samples and in individual comparisons (right) between responder and non-responder subgroups. Shared DEGs (Common) identified from unpaired (upper) and pairwise (lower) comparisons and DEGs only seen (Unique) in unpaired comparison are color-coded and plotted (left). Common DEGs seen in responder (upper) and non-responder (lower) subgroups and Unique DEGs in responders are color-coded and plotted (right). Expression changes of eight genes as annotated in volcano plots were confirmed by qRT-PCR. (C) Venn diagram showing the overlap of DEGs across each comparison pairs: overall comparison (Common genes), responder and non-responder subgroups. vs., versus; DEGs, differently expressed genes; Res, responders; Non-res, non-responders.

Supplementary Material 2: figure S2 Forest plots showing the standardized mean of gene expression difference between normal and tumor tissue as estimated from multiple studies (collected from LCE database). The leftmost column shows the included studies by the first author’s name and publication year and followed by the cohort size. The circles lined up in each column represent the effect estimates from individual studies and the very bottom circles show the pooled result for each gene as annotated. The size of each circle indicates the cohort size of individual study. The horizontal lines through the boxes illustrate the length of the 95% confidence interval in both positive and negative sides. Random-effects model was utilized to evaluate the overall effect as described by z-score and *p* value. v, versus; N, normal lung tissue; T, lung cancer tissue.

Supplementary Material 3: figure S3 (A) The hierarchical clustering of all study samples according to the profiles of responder-specific Unique DEGs identified comparing on- versus pre-treatment blood samples. The heatmap visualized the relative expression level of each DEG. Sample status (healthy control, responder etc.) are color-coded and annotated. (B) Venn diagrams and volcano plots of DEGs identified in comparing pre-treatment blood samples of responder and non-responder to healthy control (HC), respectively. Shared DEGs (Common) identified from both comparisons and DEGs only seen (Unique) in non-responders versus HCs are color-coded and plotted. (C) Principal Component Analysis (PCA) plots of healthy control samples grouped by its source (43 new from GSE datasets and 5 original donors). The left panel shows raw RNAseq data before batch effect correction while the right panel shows pre-processed data after batch effect correction. (D) PCA plots of all sample groups reported in this study. (E) Venn diagrams and volcano plots of DEGs identified in comparing on-treatment blood samples of responder and non-responder to healthy control (HC), respectively. Shared DEGs (Common) identified from both comparisons and DEGs only seen (Unique) in responders versus HCs are color-coded and plotted. (F) Venn diagrams and volcano plots of DEGs identified in comparing on-treatment blood samples of responder and non-responder to healthy control (HC), respectively. Shared DEGs (Common) identified from both comparisons and DEGs only seen (Unique) in non-responders versus HCs are color-coded and plotted. vs., versus; DEGs, differently expressed genes; Res, responders; Non-res, non-responders; HCs, healthy controls.

Supplementary Material 4: figure S4 Bubble plots of the top 30 KEGG pathways regulated in responders (A) and non-responders (B). Bubble with bigger size stands for smaller p value and higher significance. Res, responders; Non-res, non-responders.

Supplementary Material 5: figure S5 Bubble plots of the top 20 unique GO items regulated in responders (A) and non-responders (B). Bubble with bigger size stands for smaller *p* value and higher significance. Res, responders; Non-res, non-responders.

Supplementary Material 6: figure S6 Venn diagram of the top 50 gene sets downregulated (upper) or upregulated (lower) identified in comparison of on- versus pre-treatment samples. The number of unique gene sets are colored in red (responders) or blue (non-responders) while the shared gene sets are annotated in grey text. DEGs, differently expressed genes; Res, responders; Non-res, non-responders.

Supplementary Material 7: figure S7 Bubble plots of top 20 gene sets downregulated (A) and upregulated (B) in non-responders. Bubble with bigger size stands for higher k/K value ratio and larger fraction of gene was matched with a certain reference gene set.

Supplementary Material 8: figure S8 Immune cell abundance scores computed by AImmune. (A) Dot plot showing AImmune cell abundance scores of 10 immune cell subsets across five study groups as color-coded and annotated. (B) Immune cell subsets with AImmune scores that are significantly (*p* < 0.05) different across on- vs. pre-treatment samples. Pre_NS, pre-treatment samples from non-responders; On_NS, on-treatment samples from non-responders; Pre_RS, pre-treatment samples from responders; On_RS, on-treatment from responders; HCs, healthy controls; CD4, CD4 + T cells; CD8, CD8 + T cells; B, B cells; NK, natural killer cells; NKT, natural killer T cells; DC, dendritic cells; DC_ac, activated dendritic cells; Macro, macrophages; Macro_ac, activated macrophages; Mon, monocytes. All *p* values were calculated via pairwise comparisons.

Supplementary Material 9: figure S9 Immune cell fractions estimated by CIBERSORTx. (A) Stacked bar plot showing individual fractions of 22 immune cell subsets in five study groups color-coded and annotated. The different conditions are shown in different colors. (B) Immune cell subsets with CIBERSORTx fractions that are significantly (*p* < 0.05) different across on- vs. pre-treatment samples. Pre_NS, pre-treatment samples from non-responders; On_NS, on-treatment samples from non-responders; Pre_RS, pre-treatment samples from responders; On_RS, on-treatment samples from responders; HCs, healthy controls. All *p* values were calculated via pairwise comparisons.

Supplementary Material 10: figure S10 Pie charts showing distribution and counts of the reported mutations grouped by sequence ontology as identified in responders (A) and non-responders (B).

Supplementary Material 11: figure S11 A) Venn diagram comparing the mutated genes identified from the non-responders in discovery cohort versus the mutated genes from non-responder patients (those with progressive disease) of an independent cohort with cervical cancer patients (published dataset). All shared genes are top-ranked in discover cohort (bold and blue in Fig. 6B). B & C) Patients were stratified into 2 subgroups according to 1 mutational marker set (non-responder set). The Kaplan Meier overall survival curves of non-responder and unaltered subgroups. The Cox regression results indicate hazard ratios of 1.438 (95% confidence interval: 1.092 to 1.896) for non-responders in the overall patient group and 1.496 (95% confidence interval: 1.091 to 2.051) in the PD(L)1 blockade only group. Curves were generated for all immunotherapy patients (B) and patients received anti-PD(L)1 therapy only (C). Non-res, non-responders.

Supplementary Material 12: figure S12 (A) Venn diagrams and volcano plots of DEGs identified by comparing treated cells versus untreated cells. Shared DEGs (Common) identified from both cell lines and DEGs only seen (Unique) in A549 cell lines are color-coded and plotted. (B) Venn diagram visualization of the significant KEGG pathways, Reactome pathways and gene ontology (GO) items identified by comparing treated cells with untreated cells. The numbers of terms exclusively regulated in HARA-B cells, A549 cells and the shared terms are provided respectively.

Supplementary Material 13: Table S1 DEG lists identified from NSCLC patients. Table S1a List of DEGs identified in unpaired comparison (all subjects), *n* = 927. Table S1b List of DEGs identified in paired comparison (all subjects), *n* = 1211. Table S1c List of DEGs identified in responders, *n* = 2298. Table S1d List of DEGs identified in non-responders, *n* = 1025. Table S1e List of DEGs (pre-treatment vs. healthy control) exclusively identified in responders, *n* = 784. Table S1f List of DEGs (pre-treatment vs. healthy control) exclusively identified in non-responders, *n* = 589. Table S1g List of DEGs (on-treatment vs. healthy control) exclusively identified in responders, *n* = 889. Table S1h List of DEGs (on-treatment vs. healthy control) exclusively identified in non-responders, *n* = 482.

Supplementary Material 14: Table S2 List of PCR primers used in this study.

Supplementary Material 15: Table S3 DEG lists identified from co-cultured lung cancer cell lines. Table S3a List of DEGs identified in HARA-B cells, *n* = 4792. Table S3b List of DEGs identified in A549 cells, *n* = 1494.

Supplementary Material 16: Table S4 KEGG pathways identified from co-cultured lung cancer cell lines. Table S4a Downregulated KEGG pathways identified in HARA-B cells (*p* < 0.05), *n* = 50. Table S4b Upregulated KEGG pathways identified in HARA-B cells (*p* < 0.05), *n* = 90. Table S4c Downregulated KEGG pathways identified in A549 cells (*p* < 0.05), *n* = 4. Table S4d Upregulated KEGG pathways identified in A549 cells (*p* < 0.05), *n* = 0.

## Data Availability

Raw and processed files for RNA sequences (FASTQ format) supporting the findings of this study have been deposited in the National Center for Biotechnology Information Gene Expression Omnibus (NCBI-GEO) under accession number GSE225620.

## Abbreviations

CDx: Companion diagnostic
CRAVAT: Cancer-Related Analysis of Variants Toolkit
CHASM: Cancer-Specific High-Throughput Annotation of Somatic Mutations
CTLA-4: Cytotoxic T lymphocyte antigen 4
DGEs: Differentially expressed genes
DMEM: Dulbecco’s modified Eagle’s medium
DNN: Deep neural networks
ELISA: Enzyme-linked immunosorbent assay
GO: Gene ontology
GATK: Genome Analysis Toolkit v4.0
GSEA: Gene set enrichment analysis
ICIs: Immune checkpoint inhibitors
IFN-γ: Interferon-γ
NSCLC: Non-small cell lung cancer
PD-1: Programmed Death 1
PD-L1: Programmed Death-Ligand 1
pCR: Pathological complete response
qRT-PCR: Quantitative real-time PCR
RNAseq: RNA sequencing
scRNA: seqsingle-cell RNA sequencing
SqCC: Lung squamous cell carcinoma
Treg cell: Regulatory T cell
TMB: Tumor mutation burden
VEST: Variant Effect Scoring Tool
