# Supplementary figures and images for: Blood-based molecular and cellular biomarkers of early response to neoadjuvant PD-1 blockade in patients with non-small cell lung cancer
Source: Cancer Cell Int. 2024 Jun 29;24:225. doi: 10.1186/s12935-024-03412-3 (PMC11218110; doi:10.1186/s12935-024-03412-3)

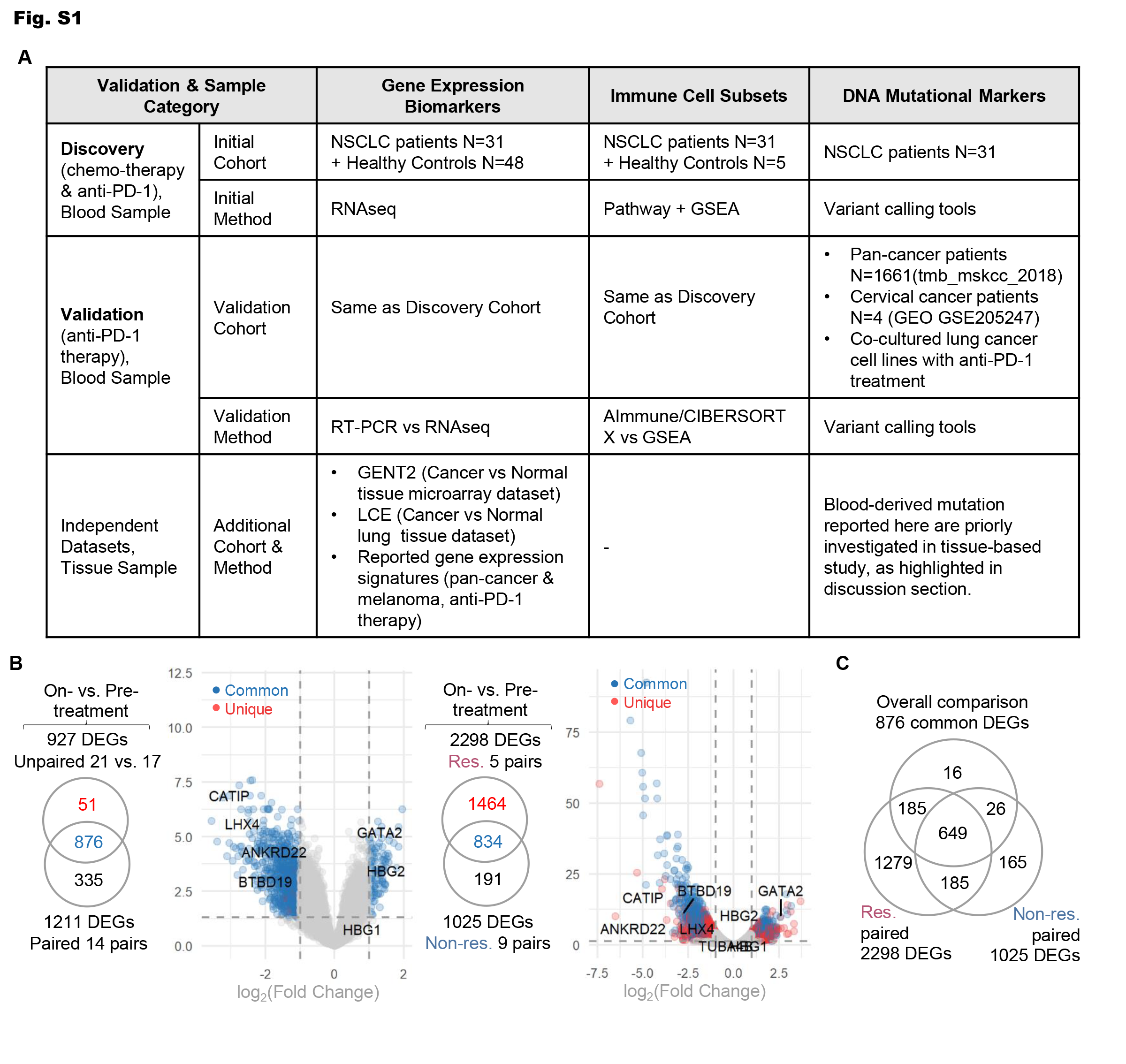

Supplement: Supplementary file 1 — Supplementary Material 1: figure S1 (A) Summary of validation cohorts, validation methods and additional datasets used in this study. (B) Venn diagrams and volcano plots of DEGs identified in overall comparisons (left) of on- versus pre-treatment blood samples and in individual comparisons (right) between responder and non-responder subgroups. Shared DEGs (Common) identified from unpaired (upper) and pairwise (lower) comparisons and DEGs only seen (Unique) in unpaired comparison are color-coded and plotted (left). Common DEGs seen in responder (upper) and non-responder (lower) subgroups and Unique DEGs in responders are color-coded and plotted (right). Expression changes of eight genes as annotated in volcano plots were confirmed by qRT-PCR. (C) Venn diagram showing the overlap of DEGs across each comparison pairs: overall comparison (Common genes), responder and non-responder subgroups. vs., versus; DEGs, differently expressed genes; Res, responders; Non-res, non-responders. [file 12935_2024_3412_MOESM1_ESM.tif]

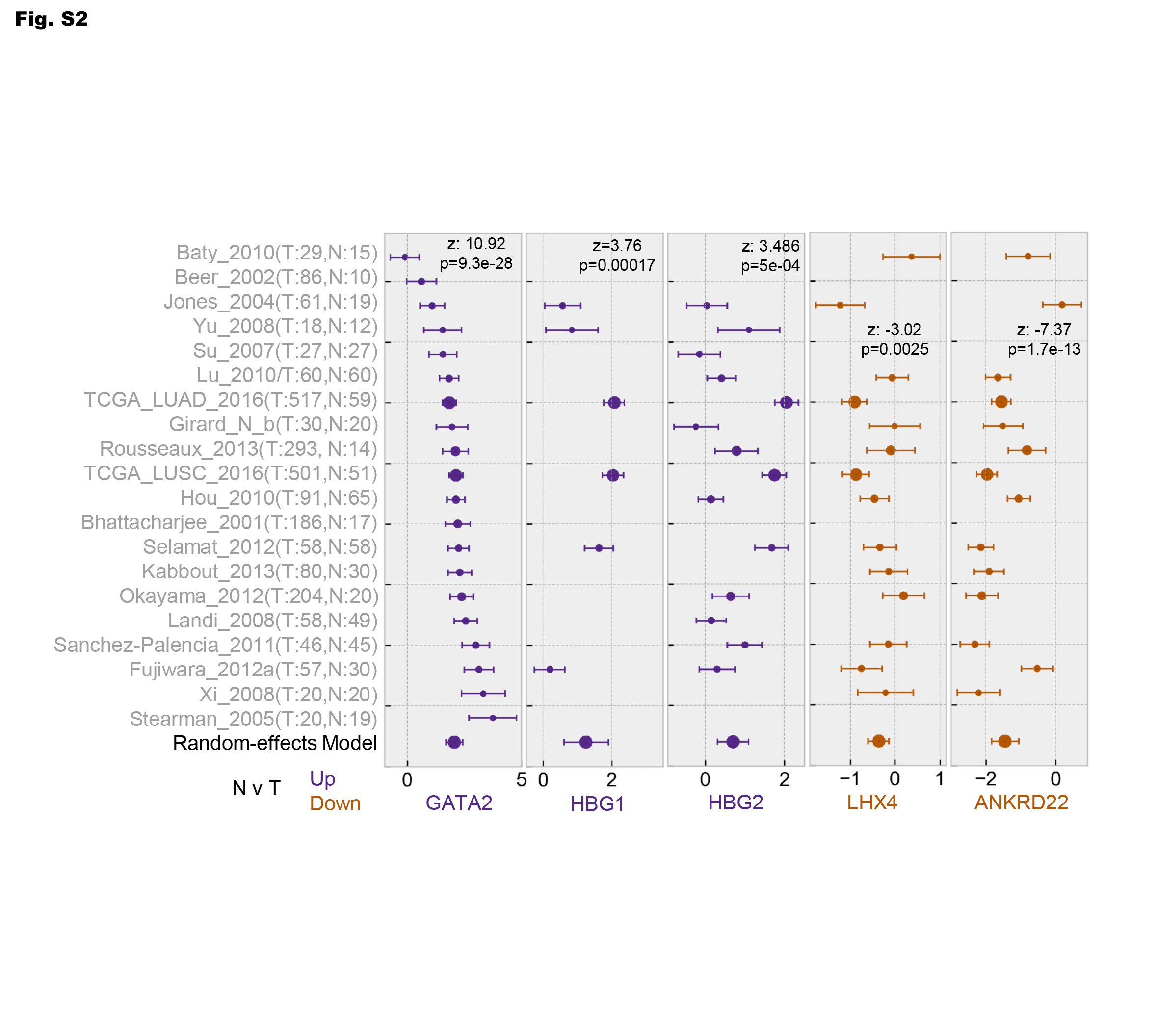

Supplement: Supplementary file 2 — Supplementary Material 2: figure S2 Forest plots showing the standardized mean of gene expression difference between normal and tumor tissue as estimated from multiple studies (collected from LCE database). The leftmost column shows the included studies by the first author’s name and publication year and followed by the cohort size. The circles lined up in each column represent the effect estimates from individual studies and the very bottom circles show the pooled result for each gene as annotated. The size of each circle indicates the cohort size of individual study. The horizontal lines through the boxes illustrate the length of the 95% confidence interval in both positive and negative sides. Random-effects model was utilized to evaluate the overall effect as described by z-score and p value. v, versus; N, normal lung tissue; T, lung cancer tissue. [file 12935_2024_3412_MOESM2_ESM.tif]

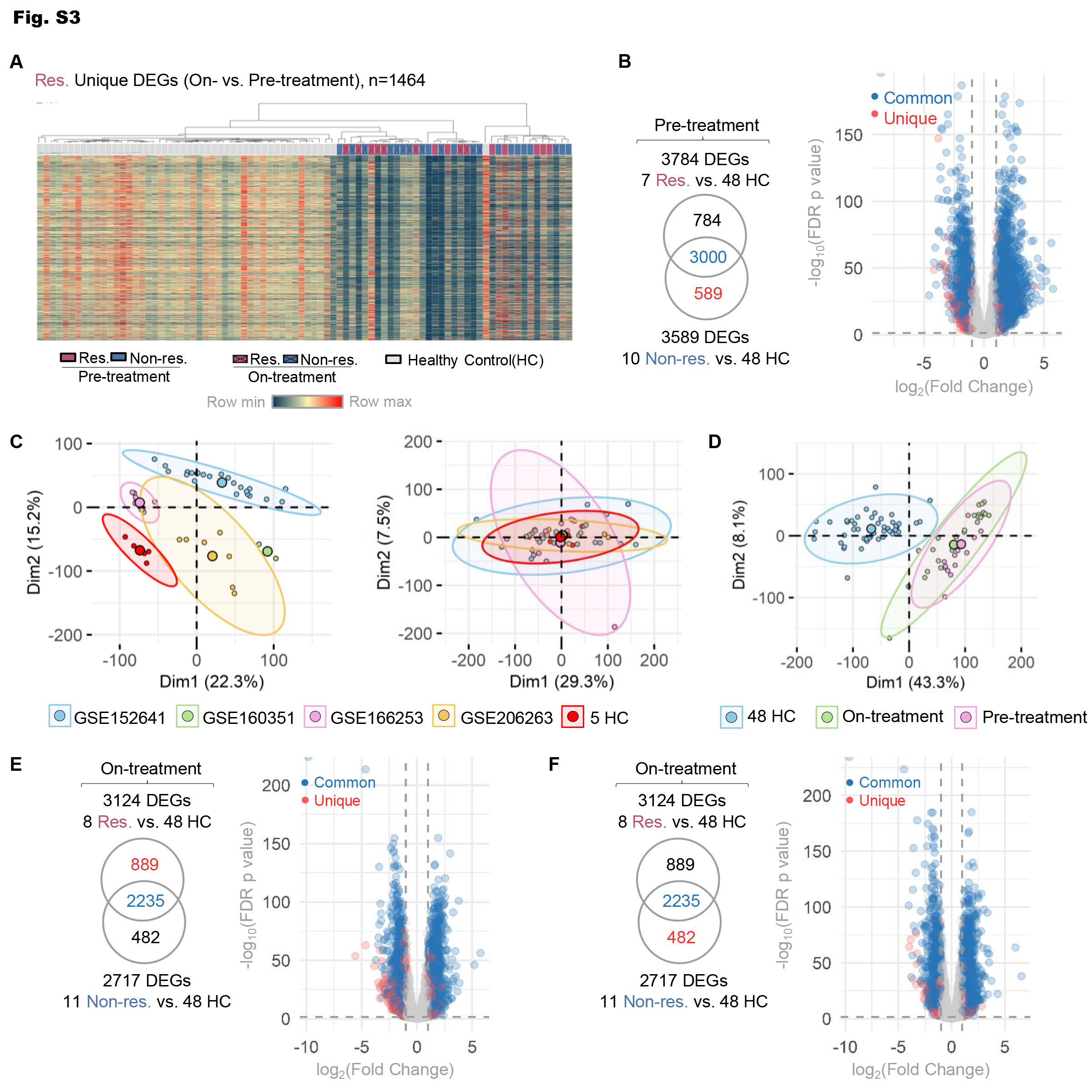

Supplement: Supplementary file 3 — Supplementary Material 3: figure S3 (A) The hierarchical clustering of all study samples according to the profiles of responder-specific Unique DEGs identified comparing on- versus pre-treatment blood samples. The heatmap visualized the relative expression level of each DEG. Sample status (healthy control, responder etc.) are color-coded and annotated. (B) Venn diagrams and volcano plots of DEGs identified in comparing pre-treatment blood samples of responder and non-responder to healthy control (HC), respectively. Shared DEGs (Common) identified from both comparisons and DEGs only seen (Unique) in non-responders versus HCs are color-coded and plotted. (C) Principal Component Analysis (PCA) plots of healthy control samples grouped by its source (43 new from GSE datasets and 5 original donors). The left panel shows raw RNAseq data before batch effect correction while the right panel shows pre-processed data after batch effect correction. (D) PCA plots of all sample groups reported in this study. (E) Venn diagrams and volcano plots of DEGs identified in comparing on-treatment blood samples of responder and non-responder to healthy control (HC), respectively. Shared DEGs (Common) identified from both comparisons and DEGs only seen (Unique) in responders versus HCs are color-coded and plotted. (F) Venn diagrams and volcano plots of DEGs identified in comparing on-treatment blood samples of responder and non-responder to healthy control (HC), respectively. Shared DEGs (Common) identified from both comparisons and DEGs only seen (Unique) in non-responders versus HCs are color-coded and plotted. vs., versus; DEGs, differently expressed genes; Res, responders; Non-res, non-responders; HCs, healthy controls. [file 12935_2024_3412_MOESM3_ESM.tif]

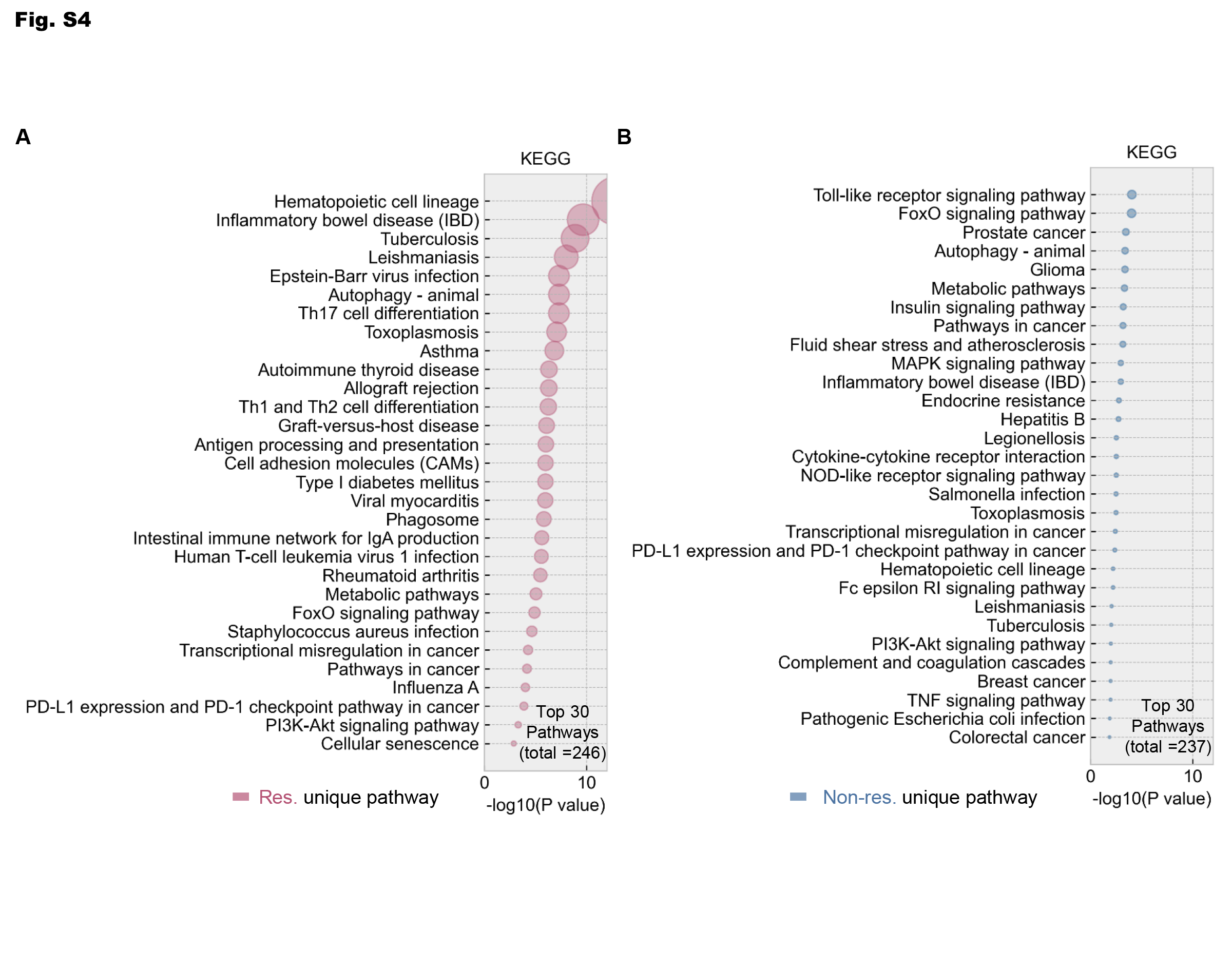

Supplement: Supplementary file 4 — Supplementary Material 4: figure S4 Bubble plots of the top 30 KEGG pathways regulated in responders (A) and non-responders (B). Bubble with bigger size stands for smaller p value and higher significance. Res, responders; Non-res, non-responders. [file 12935_2024_3412_MOESM4_ESM.tif]

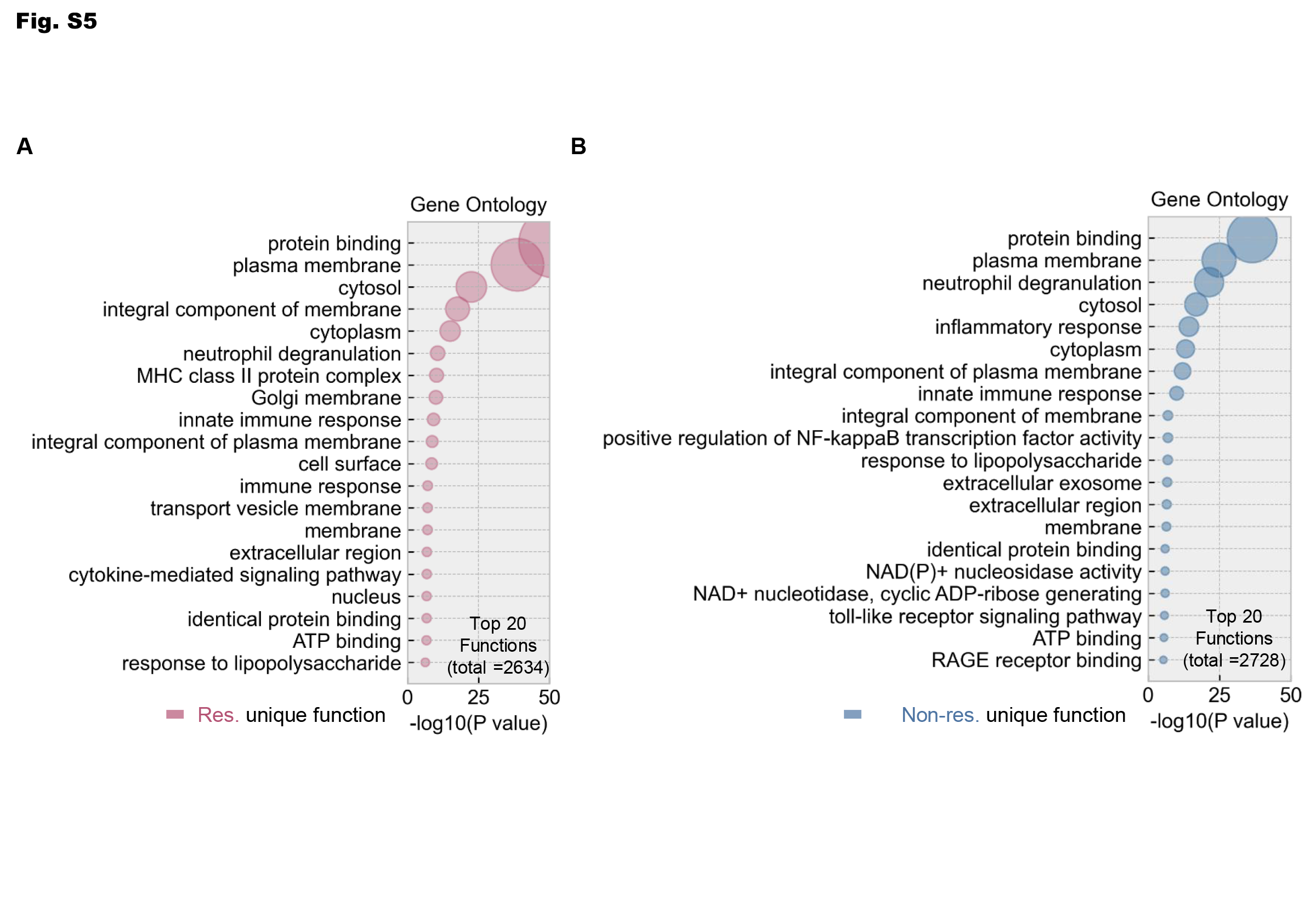

Supplement: Supplementary file 5 — Supplementary Material 5: figure S5 Bubble plots of the top 20 unique GO items regulated in responders (A) and non-responders (B). Bubble with bigger size stands for smaller p value and higher significance. Res, responders; Non-res, non-responders. [file 12935_2024_3412_MOESM5_ESM.tif]

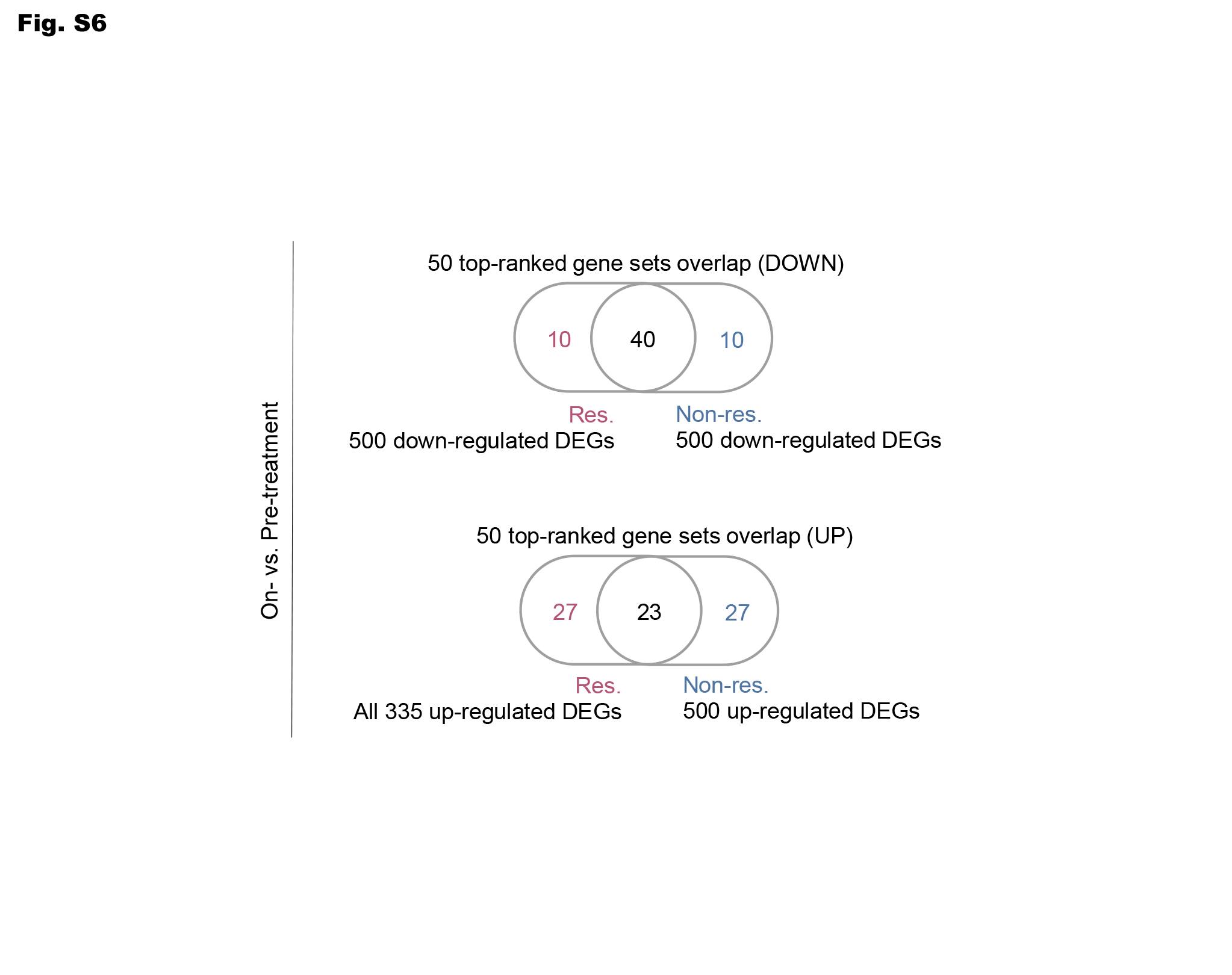

Supplement: Supplementary file 6 — Supplementary Material 6: figure S6 Venn diagram of the top 50 gene sets downregulated (upper) or upregulated (lower) identified in comparison of on- versus pre-treatment samples. The number of unique gene sets are colored in red (responders) or blue (non-responders) while the shared gene sets are annotated in grey text. DEGs, differently expressed genes; Res, responders; Non-res, non-responders. [file 12935_2024_3412_MOESM6_ESM.tif]

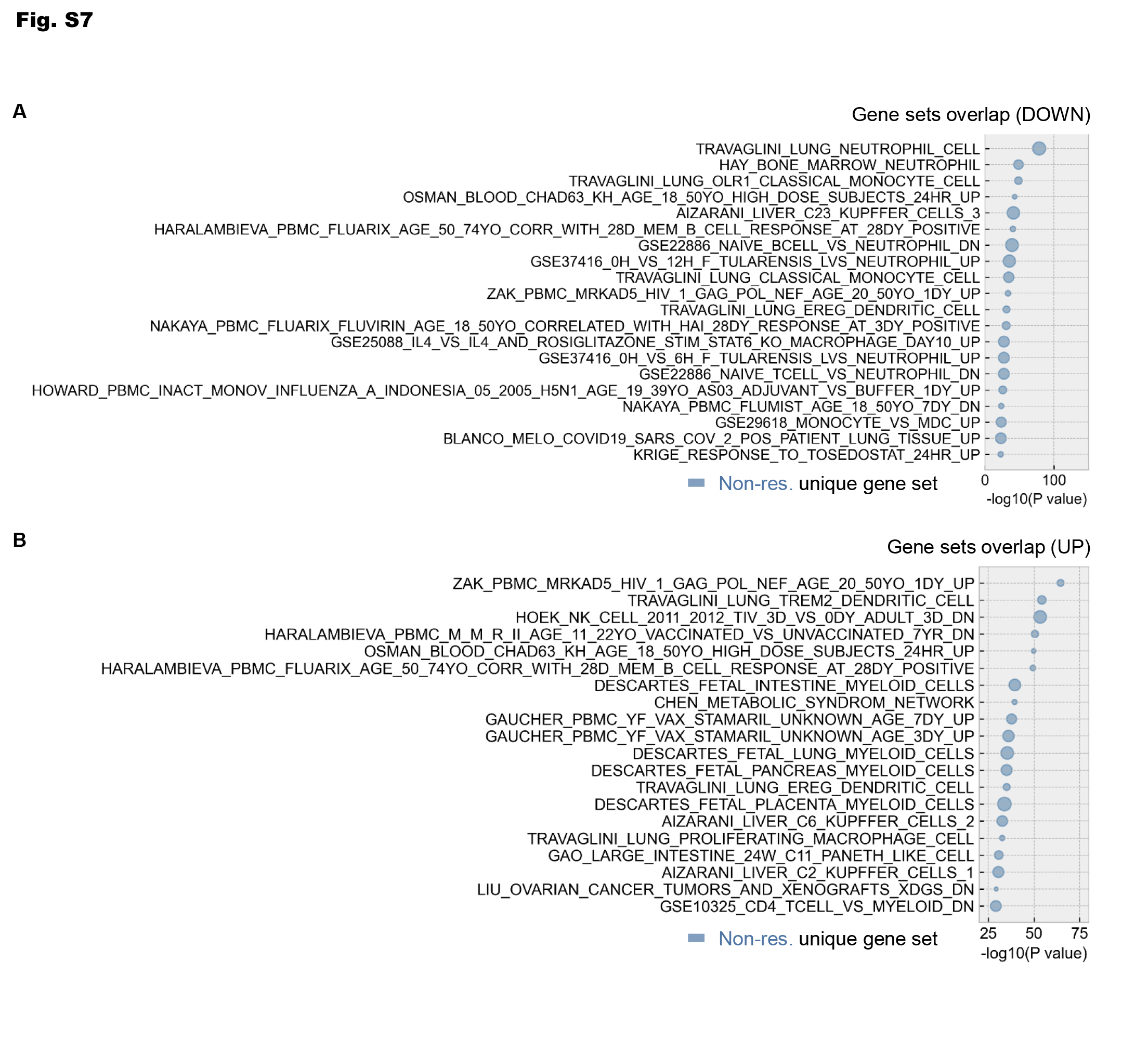

Supplement: Supplementary file 7 — Supplementary Material 7: figure S7 Bubble plots of top 20 gene sets downregulated (A) and upregulated (B) in non-responders. Bubble with bigger size stands for higher k/K value ratio and larger fraction of gene was matched with a certain reference gene set. [file 12935_2024_3412_MOESM7_ESM.tif]

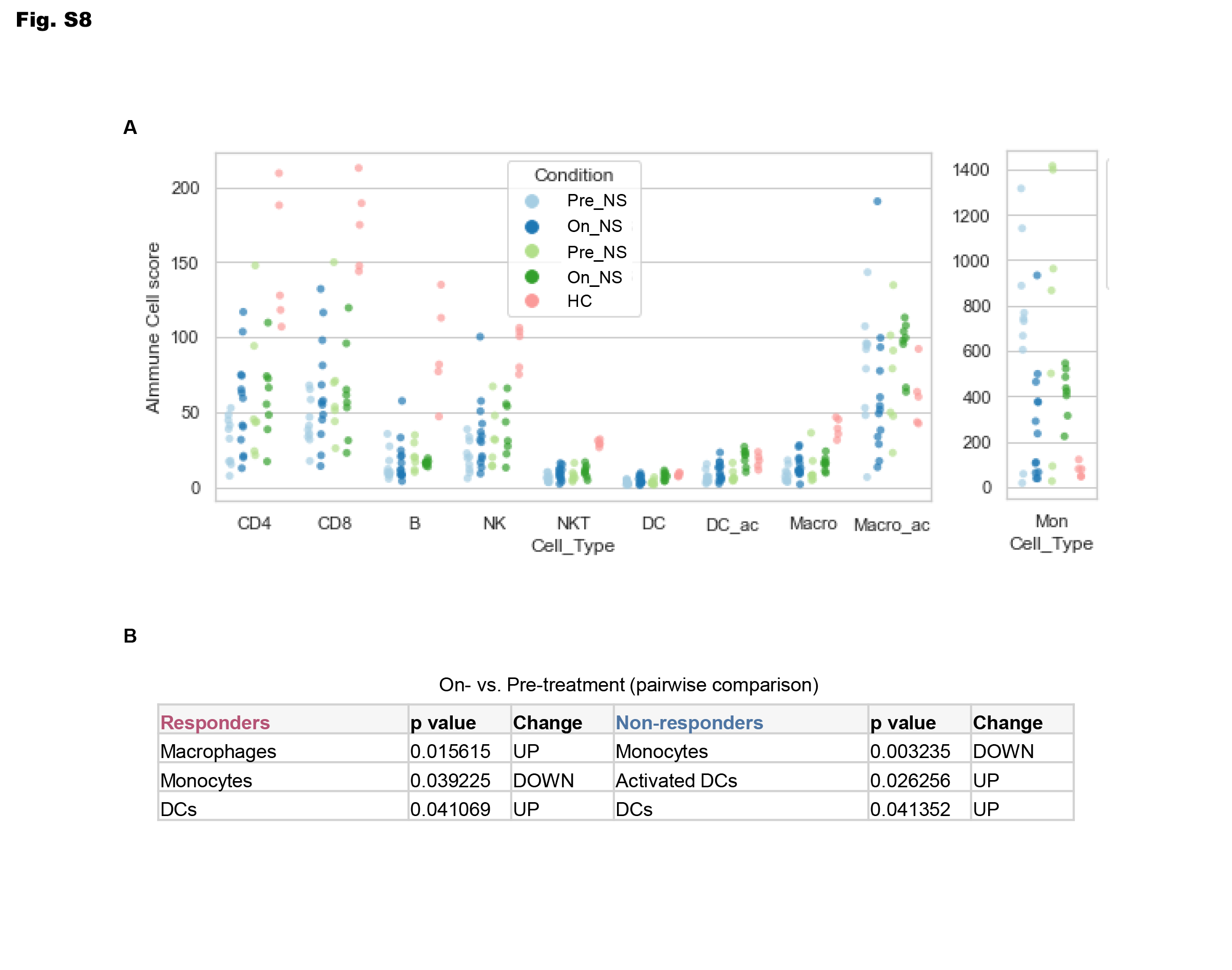

Supplement: Supplementary file 8 — Supplementary Material 8: figure S8 Immune cell abundance scores computed by AImmune. (A) Dot plot showing AImmune cell abundance scores of 10 immune cell subsets across five study groups as color-coded and annotated. (B) Immune cell subsets with AImmune scores that are significantly (p < 0.05) different across on- vs. pre-treatment samples. Pre_NS, pre-treatment samples from non-responders; On_NS, on-treatment samples from non-responders; Pre_RS, pre-treatment samples from responders; On_RS, on-treatment from responders; HCs, healthy controls; CD4, CD4 + T cells; CD8, CD8 + T cells; B, B cells; NK, natural killer cells; NKT, natural killer T cells; DC, dendritic cells; DC_ac, activated dendritic cells; Macro, macrophages; Macro_ac, activated macrophages; Mon, monocytes. All p values were calculated via pairwise comparisons. [file 12935_2024_3412_MOESM8_ESM.tif]

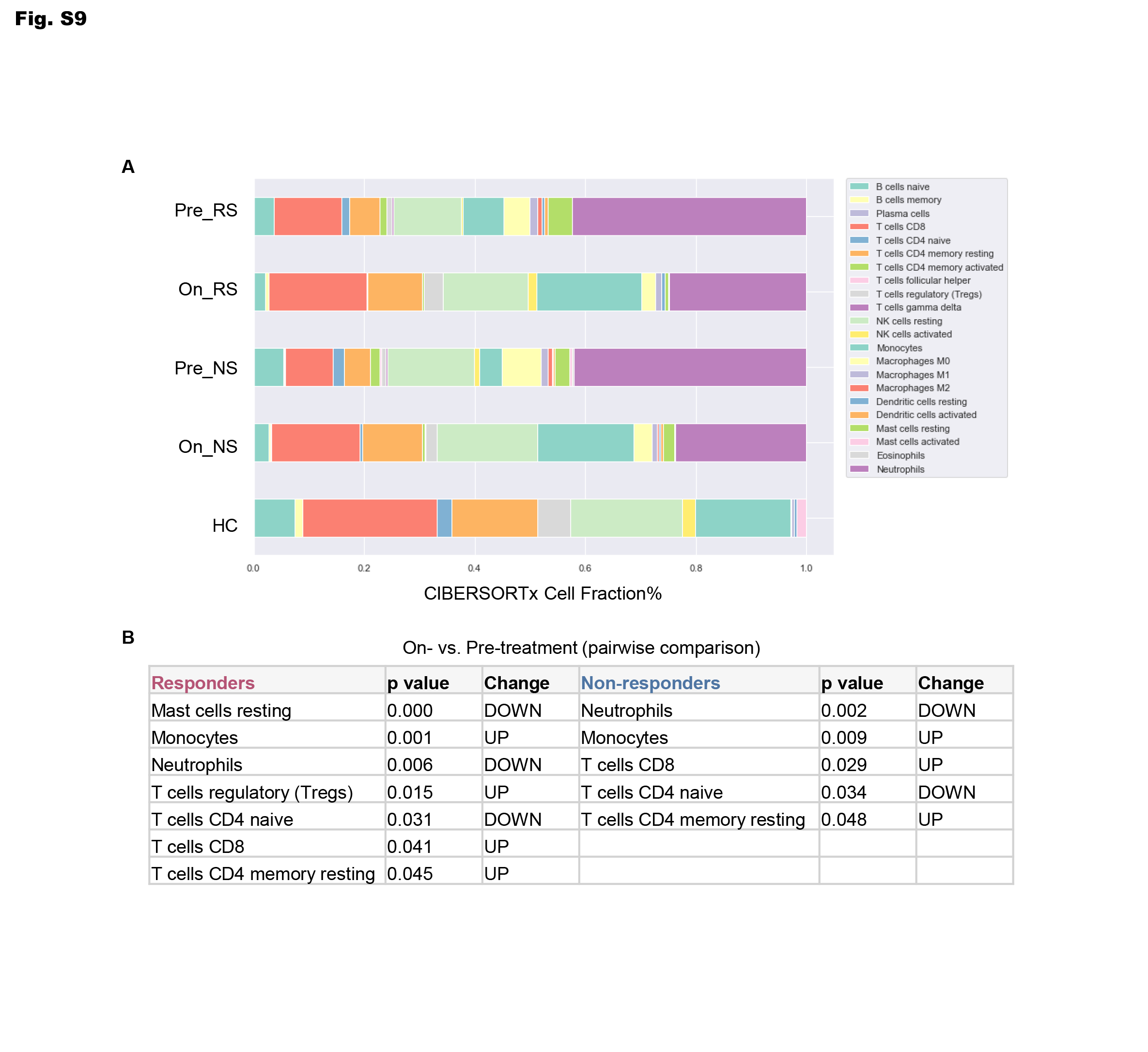

Supplement: Supplementary file 9 — Supplementary Material 9: figure S9 Immune cell fractions estimated by CIBERSORTx. (A) Stacked bar plot showing individual fractions of 22 immune cell subsets in five study groups color-coded and annotated. The different conditions are shown in different colors. (B) Immune cell subsets with CIBERSORTx fractions that are significantly (p < 0.05) different across on- vs. pre-treatment samples. Pre_NS, pre-treatment samples from non-responders; On_NS, on-treatment samples from non-responders; Pre_RS, pre-treatment samples from responders; On_RS, on-treatment samples from responders; HCs, healthy controls. All p values were calculated via pairwise comparisons. [file 12935_2024_3412_MOESM9_ESM.tif]

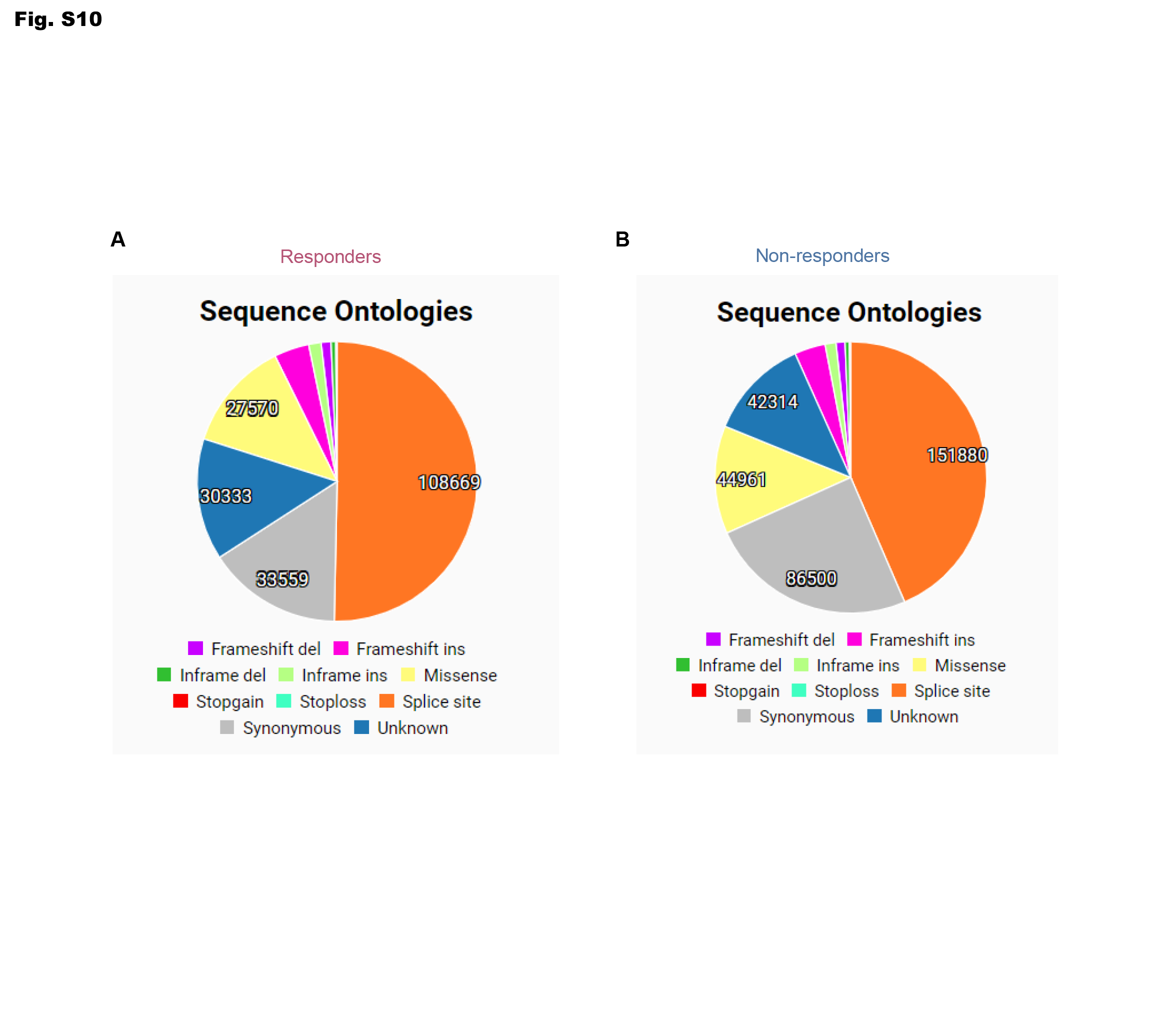

Supplement: Supplementary file 10 — Supplementary Material 10: figure S10 Pie charts showing distribution and counts of the reported mutations grouped by sequence ontology as identified in responders (A) and non-responders (B). [file 12935_2024_3412_MOESM10_ESM.tif]

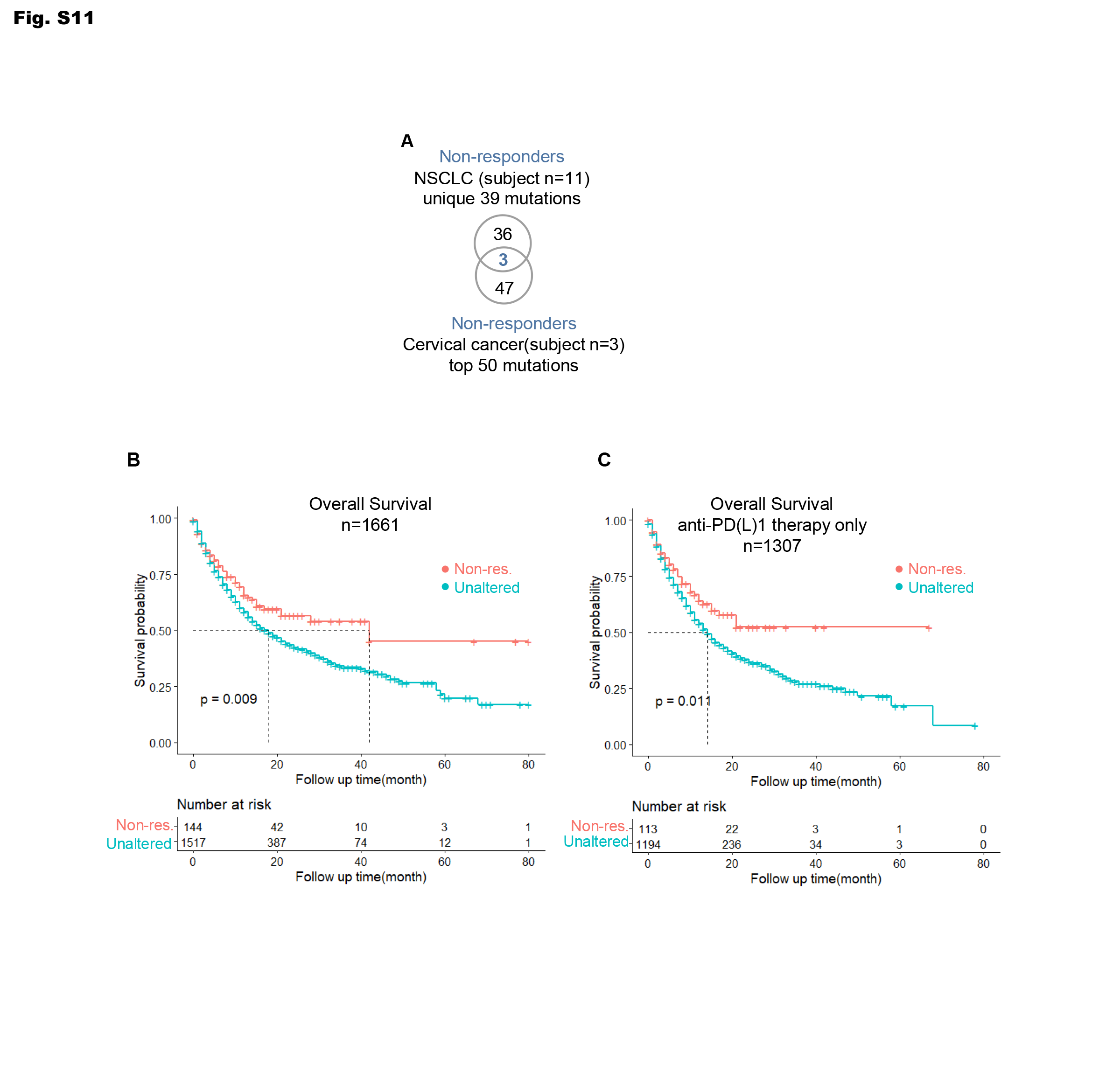

Supplement: Supplementary file 11 — Supplementary Material 11: figure S11 A) Venn diagram comparing the mutated genes identified from the non-responders in discovery cohort versus the mutated genes from non-responder patients (those with progressive disease) of an independent cohort with cervical cancer patients (published dataset). All shared genes are top-ranked in discover cohort (bold and blue in Fig. 6B). B & C) Patients were stratified into 2 subgroups according to 1 mutational marker set (non-responder set). The Kaplan Meier overall survival curves of non-responder and unaltered subgroups. The Cox regression results indicate hazard ratios of 1.438 (95% confidence interval: 1.092 to 1.896) for non-responders in the overall patient group and 1.496 (95% confidence interval: 1.091 to 2.051) in the PD(L)1 blockade only group. Curves were generated for all immunotherapy patients (B) and patients received anti-PD(L)1 therapy only (C). Non-res, non-responders. [file 12935_2024_3412_MOESM11_ESM.tif]

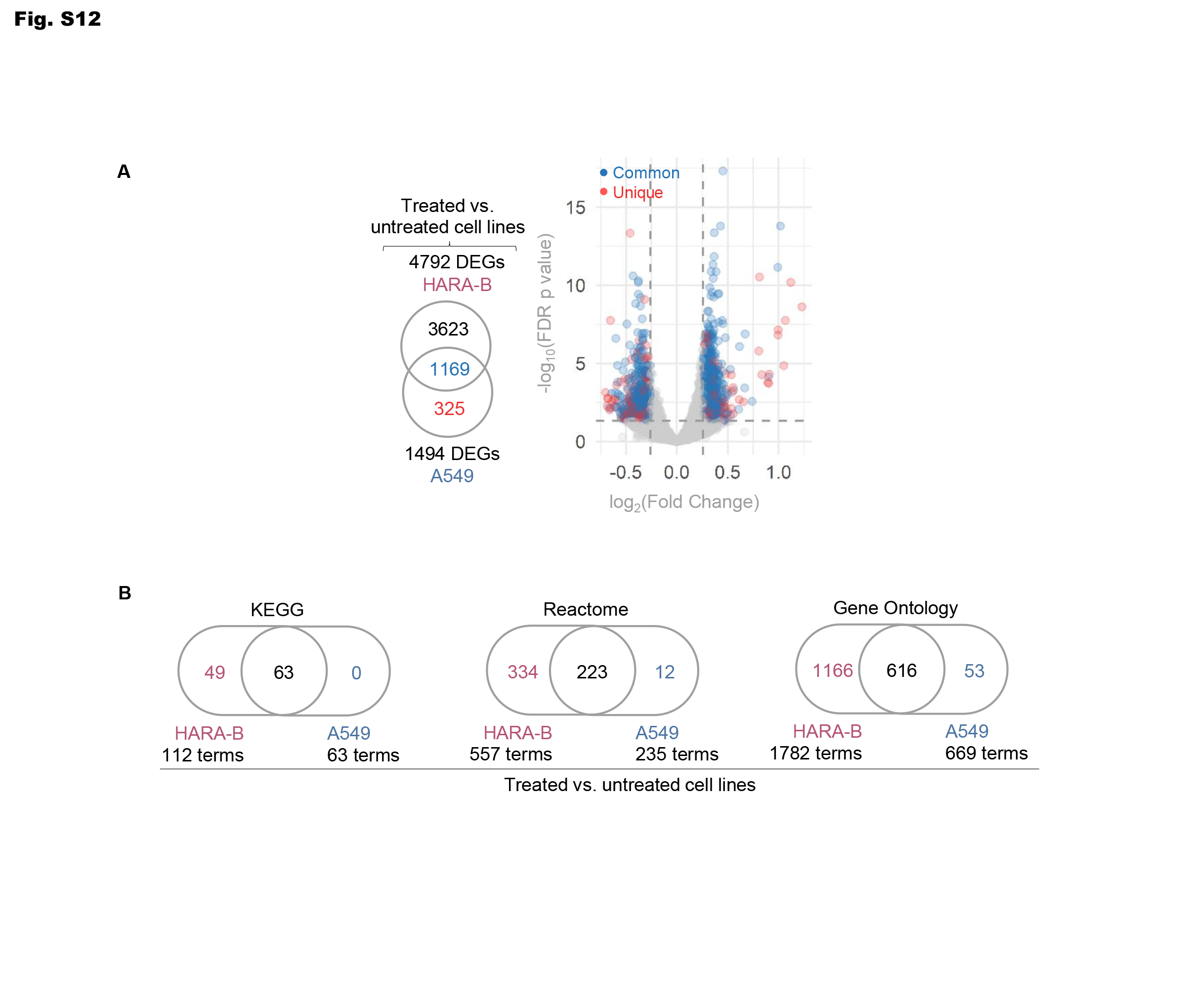

Supplement: Supplementary file 12 — Supplementary Material 12: figure S12 (A) Venn diagrams and volcano plots of DEGs identified by comparing treated cells versus untreated cells. Shared DEGs (Common) identified from both cell lines and DEGs only seen (Unique) in A549 cell lines are color-coded and plotted. (B) Venn diagram visualization of the significant KEGG pathways, Reactome pathways and gene ontology (GO) items identified by comparing treated cells with untreated cells. The numbers of terms exclusively regulated in HARA-B cells, A549 cells and the shared terms are provided respectively. [file 12935_2024_3412_MOESM12_ESM.tif]
